# Transferability of Type 2 Diabetes Implicated Loci in Multi-Ethnic Cohorts from Southeast Asia

**DOI:** 10.1371/journal.pgen.1001363

**Published:** 2011-04-07

**Authors:** Xueling Sim, Rick Twee-Hee Ong, Chen Suo, Wan-Ting Tay, Jianjun Liu, Daniel Peng-Keat Ng, Michael Boehnke, Kee-Seng Chia, Tien-Yin Wong, Mark Seielstad, Yik-Ying Teo, E-Shyong Tai

**Affiliations:** 1 Centre for Molecular Epidemiology, National University of Singapore, Singapore, Singapore; 2 NUS Graduate School for Integrative Science and Engineering, National University of Singapore, Singapore, Singapore; 3 Genome Institute of Singapore, Agency for Science, Technology and Research, Singapore, Singapore; 4 Singapore Eye Research Institute, Singapore National Eye Centre, Singapore, Singapore; 5 Department of Epidemiology and Public Health, National University of Singapore, Singapore, Singapore; 6 Department of Biostatistics and Center for Statistical Genetics, School of Public Health, University of Michigan, Ann Arbor, Michigan, United States of America; 7 Department of Ophthalmology, National University of Singapore, Singapore, Singapore; 8 Centre for Eye Research Australia, University of Melbourne, Melbourne, Australia; 9 Department of Statistics and Applied Probability, National University of Singapore, Singapore, Singapore; 10 Department of Medicine, National University of Singapore, Singapore, Singapore; 11 Duke-National University of Singapore Graduate Medical School, Singapore, Singapore; Georgia Institute of Technology, United States of America

## Abstract

Recent large genome-wide association studies (GWAS) have identified multiple loci which harbor genetic variants associated with type 2 diabetes mellitus (T2D), many of which encode proteins not previously suspected to be involved in the pathogenesis of T2D. Most GWAS for T2D have focused on populations of European descent, and GWAS conducted in other populations with different ancestry offer a unique opportunity to study the genetic architecture of T2D. We performed genome-wide association scans for T2D in 3,955 Chinese (2,010 cases, 1,945 controls), 2,034 Malays (794 cases, 1,240 controls), and 2,146 Asian Indians (977 cases, 1,169 controls). In addition to the search for novel variants implicated in T2D, these multi-ethnic cohorts serve to assess the transferability and relevance of the previous findings from European descent populations in the three major ethnic populations of Asia, comprising half of the world's population. Of the SNPs associated with T2D in previous GWAS, only variants at *CDKAL1* and *HHEX/IDE*/*KIF11* showed the strongest association with T2D in the meta-analysis including all three ethnic groups. However, consistent direction of effect was observed for many of the other SNPs in our study and in those carried out in European populations. Close examination of the associations at both the *CDKAL1* and *HHEX/IDE/KIF11* loci provided some evidence of locus and allelic heterogeneity in relation to the associations with T2D. We also detected variation in linkage disequilibrium between populations for most of these loci that have been previously identified. These factors, combined with limited statistical power, may contribute to the failure to detect associations across populations of diverse ethnicity. These findings highlight the value of surveying across diverse racial/ethnic groups towards the fine-mapping efforts for the casual variants and also of the search for variants, which may be population-specific.

## Author Summary

Type 2 diabetes mellitus (T2D) is a chronic disease which can lead to complications such as heart disease, stroke, hypertension, blindness due to diabetic retinopathy, amputations from peripheral vascular diseases, and kidney disease from diabetic nephropathy. The increasing prevalence and complications of T2D are likely to increase the health and economic burden of individuals, families, health systems, and countries. Our study carried out in three major Asian ethnic groups (Chinese, Malays, and Indians) in Singapore suggests that the findings of studies carried out in populations of European ancestry (which represents most studies to date) may be relevant to populations in Asia. However, our study also raises the possibility that different genes, and within the genes different variants, may confer susceptibility to T2D in these populations. These findings are particularly relevant in Asia, where the greatest growth of T2D is expected in the coming years, and emphasize the importance of studying diverse populations when trying to localize the regions of the genome associated with T2D. In addition, we may need to consider novel methods for combining data across populations.

## Introduction

Type 2 diabetes mellitus (T2D) is a major chronic disease worldwide, affecting more than 300 million people. The greatest increase in the prevalence of T2D in the coming years is likely to be in Asia, home to half of the world's population with 3 billion people [1]–[2]. It is estimated that in China alone, there are 100 million people with T2D [3].

T2D has been one of the major human diseases to benefit from the advent of large-scale genetic studies that survey the entire genomic landscape for variants correlating with disease onset or severity. These genome-wide association studies (GWAS) have identified a number of novel loci harboring common variants that are associated with an increased risk of T2D [4]–[11], adding to the loci previously identified by candidate gene studies (*PPARG* [12], *KCNJ11* [13]–[14], *WFS1* [15]–[16]) and linkage studies *TCF7L2* [17]–[19]). There have been fewer GWAS of T2D performed in non-European populations, namely in the Japanese [20], Han Chinese in Taiwan [21] and South Asians in the United Kingdom [22]. These latter studies, however, are important for the following reasons: (i) the frequencies of genuinely implicated variants may differ across ethnic groups, and these studies may discover novel regions that have been overlooked in previous studies due to lower risk allele frequencies in populations of European ancestry; (ii) ethnicity may modulate the associations between common variants and T2D, such that the same locus may exert different effects in other populations due to differences in genetic background or environmental exposures; (iii) the pathogenesis of T2D may be heterogeneous across populations, resulting in differing importance of genetic susceptibility to a particular loci. Examples of novel findings emerging from the Asian GWAS include variants in *KCNQ1* [8]–[9], *PTPRD* [21], *SRR* [21] and *PEPD* [20]. Of these, only the association at *KCNQ1* has been extensively replicated [11], [20]–[21], [23]–[30]. The discovery of these genetic loci for T2D is exciting, since it heralds the prospect of identifying novel therapeutic targets for its treatment and prevention. For example, *PPARG* and *KCNJ11* both harbor common genetic variants associated with T2D and are both therapeutic targets for drugs used to lower blood glucose [12]–[13].

However, at present, there is limited information on the relevance of the identified loci across multiple populations, as these discoveries have primarily been made in populations of European ancestry. Even if the same locus is causally implicated with T2D onset in multiple populations, it is unclear whether the genetic effect estimated from these studies is representative in other non-European populations. One common strategy of evaluating the transferability and the genetic effects of the associated loci is to replicate the index SNPs that have been identified by the genome-wide surveys. Many of these studies, conducted in individuals of Han Chinese, Japanese, Asian Indians, exhibited replication for many of the index SNPs that emerged from the first wave of T2D GWAS [4]–[7] with consistent direction of effect [20], [23]–[24], [30]–[45]. However, replication efforts for the more recently identified SNPs have been less successful [32], [46]–[48]. Failure to replicate the original associations at the index SNPs in heterogeneous populations does not necessarily indicate that these loci are not involved in T2D pathogenesis in these populations. As these SNPs are unlikely to be the biologically functional polymorphisms but merely in linkage disequilibrium (LD) with the underlying causal variants, the index SNPs identified from European populations may be poorly correlated with the causal variants in other populations such that studies aiming to reproduce only the original associations are under-powered and thus are unable to observe any statistical evidence at these SNPs. GWAS carried out in non-European populations can also address the two issues of transferability and consistency of the genetic etiology between populations with differing ancestry.

The multi-ethnic demography of Singapore, consisting mainly of Chinese, Malays and Asian Indians, possesses vast potential for investigating the genetic etiology of T2D. Importantly, these populations broadly capture the genetic diversity across Asia, home to almost three billion people, and especially in a large proportion of the populations that are likely to experience the greatest increase in the burden of T2D in the near future [1].

Here we describe three separate genome-wide surveys of T2D in the Chinese, Malay and Asian Indian populations assaying a total of 10,718 individuals, yielding a post-QC sample size of 3,781 cases and 4,354 controls. With this resource, we set out: (i) to identify any novel genetic variants that are associated with T2D in these ethnic groups; (ii) to examine the genetic architecture of the previously established T2D loci in the heterogeneous settings offered by the three ethnic groups; and (iii) to estimate the magnitude of the effects at variants that replicate in our populations.

## Results

We performed three population-based case-control GWAS in T2D in 10,718 individuals of Chinese, Malay and Asian Indian ethnicities living in Singapore. A total of 3,955 Chinese (2,010 cases, 1,945 controls), 2,034 Malays (794 cases, 1,240 controls) and 2,146 Asian Indians (977 cases, 1,169 controls) remained after sample quality control. The Chinese samples were genotyped on a combination of Illumina610 and Illumina1M arrays, while the Malays and Indians were entirely genotyped on the Illumina610 array (Figure S1). In general, cases were older than controls and the Malays and Indians were more obese than Chinese irrespective of case-control status. Amongst the Chinese, there were more men genotyped on the Illumina1M array than on Illumina610 (Table 1). The genomic inflation factors were 1.049 for Chinese on the Illumina610 array, 1.058 for Chinese on the Illumina1M array and 1.017 for the combined Chinese. The inflation factors were 1.035 and 1.030 for the Malays and Indians respectively, with an overall genomic factor 1.007 for all populations combined.

**Table 1:** Summary characteristics of cases and controls stratified by their ethnic groups and genotyping arrays.

Characteristics	Chinese				Malay^a^		Asian Indian^a^	
	Illumina610quad		Illumina1Mduov3		Illumina610quad		Illumina610quad	
	Cases	Controls	Cases	Controls	Cases	Controls	Cases	Controls
N	1,082	1,006	928	939	794	1240	977	1169
Sex Ratio M/F (%)	402/680 (37.15/62.85)	217/789 (21.57/78.43)	602/326 (64.87/35.13)	599/340 (63.79/36.21)	405/389 (51.01/48.99)	645/595 (52.02/47.98)	531/466 (54.35/45.65)	566/603 (48.42/51.58)
Age^b^ (yr)	65.07 (9.70)	47.69 (11.07)	63.67 (10.81)	46.74 (10.23)	62.27 (9.90)	56.89 (11.39)	60.71 (9.85)	55.73 (9.72)
Age at diagnosis (yr)	55.65 (11.96)	--	52.15 (14.40)	--	54.35 (11.19)	--	51.35 (10.63)	--
Fasting glucose (mmol/L)	--	4.67 (0.45)	--	4.73 (0.46)	--	--	--	--
HbA1C	--	--	--	--	8.05 (1.84)	5.60 (0.30)	7.56 (1.52)	5.55 (0.28)
BMI^1^ (kg/m^2^)	25.27 (3.92)	22.30 (3.67)	25.42 (3.81)	22.84 (3.41)	27.82 (4.88)	25.13 (4.82)	27.06 (5.10)	25.33 (4.40)

**a** For Malay and Asian Indian samples, diabetic samples are defined as either with history of diabetes or hba1c ≥6.5% while controls are defined as no history of diabetes and hba1c<6%.

**b** Mean(Standard Error).

### Top regions emerging from genome-wide scans

The Indian GWAS identified a SNP (rs1048886) intronic to a hypothetical protein (*C6orf57*) on chromosome 6 which exhibited genome-wide significance (OR = 1.54, 95% CI = 1.32 – 1.80, *P* = 3.48×10^−8^) although this was not statistically significant in the Chinese (*P* = 0.995) or the Malays (*P* = 8.23×10^−2^). No SNP achieved genome-wide significance in the individual Chinese and Malays genome scans, or in the meta-analysis across all three populations. SNPs that exhibited suggestive evidence of association with T2D at *P*-value<10^−5^ in each ethnic group are shown in Table S1. SNPs at 6 loci showed suggestive evidence of association with T2D at *P*-value<10^−5^ after meta-analysis of the three ethnic groups (Table 2 and Table S2). These include *HMG20A*, *ZPLD1* and *HUNK* which showed no evidence of heterogeneity from *I^2^* statistics; *C6orf57* which was driven primarily by the Indians; and the well-established gene regions at *CDKAL1* and *KIF11* (Table 2, Table S2 and Figure S5). More details are provided in Text S1.

**Table 2:** Statistical evidence of the top regions (defined as *P*<10^−5^) that emerged from the fixed-effects meta-analysis of the GWAS results across Chinese, Malays, and Asian Indians, with information on whether each SNP is a directly observed genotype (1) or is imputed (0).

SNP	Chr	Pos (bp)	Nearest gene	Risk allele	Reference allele	Genotyped (1) or imputed (0)^a^	N	Chinese + Malays + Indians (3781 cases/4354 controls)			
								Risk allele frequency^b^	Fixed effects OR (95% CI)	Fixed effects *P-*value	*I* ^2^ (%)
rs7119	15	75564687	*HMG20A*	T	C	1111	8135	0.188	1.24 (1.14–1.34)	5.24×10^−7^	0
rs2063640	3	103685735	*ZPLD1*	A	C	1111	8131	0.167	1.23 (1.13–1.34)	3.47×10^−6^	0
rs2833610	21	32307057	*HUNK*	A	G	1111	8127	0.567	1.17 (1.09–1.24)	3.90×10^−6^	0
rs6583826	10	94337810	*KIF11*	G	A	1111	8134	0.259	1.18 (1.10–1.27)	7.38×10^−6^	0
rs1048886	6	71345910	*C6orf57*	G	A	1111	8135	0.110	1.26 (1.14–1.39)	9.70×10^−6^	85.40
rs9295474	6	20760696	*CKDAL1*	G	C	0000	8079	0.357	1.16 (1.09–1.24)	8.59×10^−6^	33.46

Combined minor allele frequencies of each lead SNP is at least 5%. The *I^2^* statistic refers to the test of heterogeneity of the observed odds ratios for the risk allele in the three populations, and is expressed here as a percentage.

**a** This column shows whether each SNP is directly genotyped (1) or imputed (0) in each of the case control studies shown in Table 1. Each digit represents a case control study in the following order from left to right: Chinese on Illumina610, Chinese on Illumina1M, Malays on Illumina610 and Indians on Illumina610.

**b** Risk allele frequencies are sample size weighted frequencies across the three ethnic groups.

### Evaluating transferability of known loci across populations

In assessing whether there was evidence in our GWAS scans to support the associations established in previous studies on T2D onset, we defined statistical significance as *P*-value<0.05. Even with the reduced stringency, we noticed that only the SNPs in *CDKAL1* and *HHEX/IDE/KIF11* replicated in the meta-analysis across all three populations (Table 2). While the reported index SNPs at *KCNJ11* replicated in Chinese and Malays (Table S3), the majority of the established associations in other genes were only detected in one population, including *TCF2*/*HNF1B*, *IGF2BP2*, *CENTD2*, *C2CD4A-C2CD4B* and *FTO* in the Chinese; *KCNQ1* and *PRC1* in the Malays; and *TCF7L2* and *BCL11A* in the Indians (Table S3). In addition, the meta-analysis also supported the reported associations at *IRS1* and *SLC30A8* despite none of the SNPs achieving statistical significance in the single-population analyses (Table S3).

**Figure 1:**
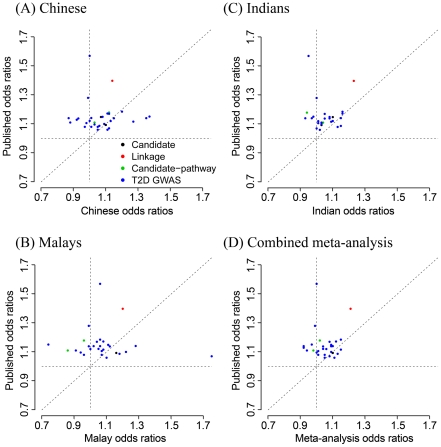
Bivariate plots comparing odds ratios observed in each of the ethnic groups with odd ratios established in populations of European ancestry. (A) Chinese, (B) Malays, (C) Indians, (D) Combined meta-analysis. Each SNP is plotted with a colour that indicates if the SNP was identified through candidate gene studies (black) or linkage studies (red) or candidate-pathway analysis (green) or T2D genome-wide scans (blue).

Our single-population analyses and meta-analysis failed to detect associations at several loci, including *PPARG*, *WFS1* and several regions that were identified through T2D GWAS in European GWAS. This may be attributed to the lower statistical power in our three GWAS as many of these regions possess only modest effects on T2D pathogenesis and have only been successfully identified in large-scale meta-analyses involving tens of thousands of samples. To detect ORs exhibited in the European ranging from 1.10 to 1.25 [11], our studies are not sufficiently powered even at risk allele frequencies of 0.30 and higher (Figure S4). Thus, in evaluating the relevance of these established findings in our populations, we took a number of approaches. Firstly, we performed a binomial test on the number of loci expected to have *P*-value less than 0.05 which showed evidence of an over-representation of the European established loci in the Chinese and Indians and combined meta-analysis with one sided *P*-values given by 2.85×10^−4^ for Chinese, 1.05×10^−01^ for Malays, 2.22×10^−02^ for Indians and 3.31×10^−07^ for Meta-analysis. Next, we observed that most of the associations observed in these genes (with the exception of *PEPD* where the risk allele initially discovered in a Japanese study [20] conferred a protective effect in our populations instead) trended in the same direction, i.e. the same allele conferred risk in all three populations as the published results, with a binomial test for consistency of direction giving the following p-values: Chinese: 5.92×10^−3^; Malay: 9.30×10^−2^; Indian: 4.34×10^−3^; Meta-analysis: 1.49×10^−3^). Finally, at lead SNPs reported in T2D studies in European populations, we also compared the detected effect sizes of the risk alleles in our study and those in European populations. Whenever possible, we used the effect sizes from stage 2 of DIAGRAM+ [11]. This approach allows us to test the hypothesis that the observed effect sizes across multiple populations should be comparable at a SNP that is genuinely associated with T2D in these populations despite limited power. There was a greater proportion of SNPs displaying attenuated odds ratios in our populations when compared to the effect sizes at the lead SNPs from DIAGRAM+ consortium [11] (with two-sided P-values given by Chinese: 5.22×10^−2^; Malay: 3.47×10^−2^; Indian: 8.55×10^−4^; Meta-analysis: 7.20×10^−3^ and Figure 1).

**Figure 2:**
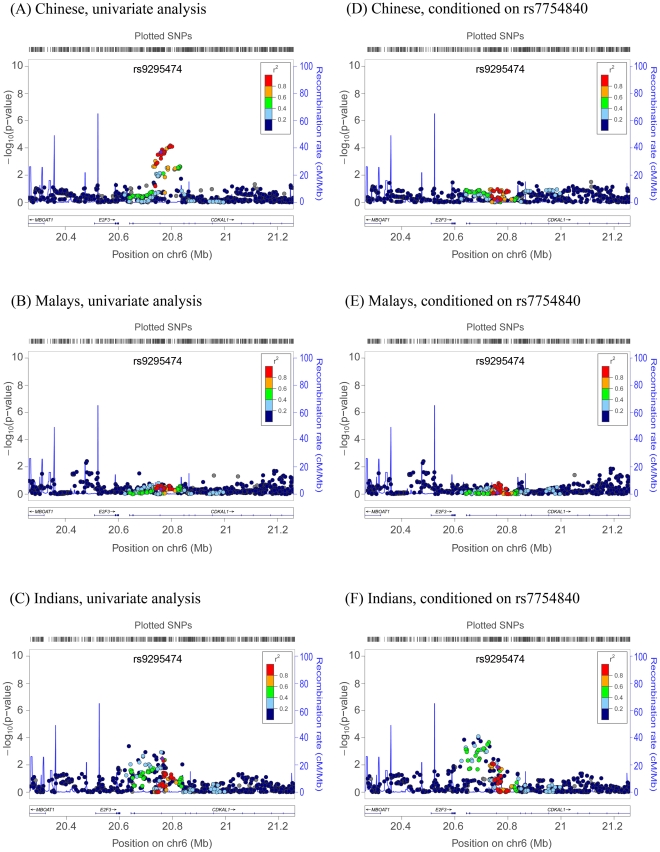
Regional association plots of the index SNP in *CDKAL1*. For each ethnic group, the univariate analysis regional plot, A) Chinese B) Malays C) Indians, is shown together with analysis conditioned on established index SNP rs7754840, D) Chinese E) Malays F) Indians, in populations of Caucasian ancestry. In each panel, the index SNP is represented with a purple diamond and surrounding SNPs coloured based on their r^2^ with the index SNP. Estimated recombination rates reflect the local LD structure in the 500kb buffer around the index SNP and the proxies are plotted on Hapmap values from the Hapmap JPT+CHB. Data for gene annotations are obtained from the RefSeq track of the UCSC Gene Browser (See LocusZoom http://csg.sph.umich.edu/locuszoom/ for more details).

The availability of three GWAS scans across three genetically heterogeneous populations offers a unique opportunity to explore the genetic architecture underlying the two loci *CDKAL1* and *HHEX*/*IDE*/*KIF11* that showed the strongest evidence of association with T2D in more than 1 population and in our meta-analysis. We observed a cluster of SNPs displaying evidence of association *P*<0.01 at the *CDKAL1* locus in both the Chinese (Figure 2A) and Indians (Figure 2C) scans. However, there was no evidence of T2D association in the Malays (Figure 2B). The top signal emerging from the meta-analysis (rs9295474, meta-analysis *P* = 8.59×10^−6^) was located at 20.761Mb on chromosome 6, and the risk allele frequency was 38.2%, 38.4% and 28.4% in the Chinese, Malay and Indian populations, respectively. We subsequently performed an analysis at this region conditioned on the top SNP (rs7754840) that emerged from T2D studies in populations of European descent. This conditional analysis effectively removed any further evidence of T2D association at this locus in the Chinese samples (Figure 2D), indicating that the observed associations in the Chinese might be attributed to the same functional polymorphism that is responsible for the association signals in Europeans. Intriguingly, the conditional analysis only partially attenuated the signal in Indians, and instead appeared to strengthen the association evidence in the upstream region of *CDKAL1*. This region was found to exhibit evidence of regional LD variation between the Indians and CEU (varLD monte carlo *P* = 1.16×10^−2^) (Figure 2F and Table S4), suggesting the possibility of different biological mechanisms or causal signals.

**Figure 3:**
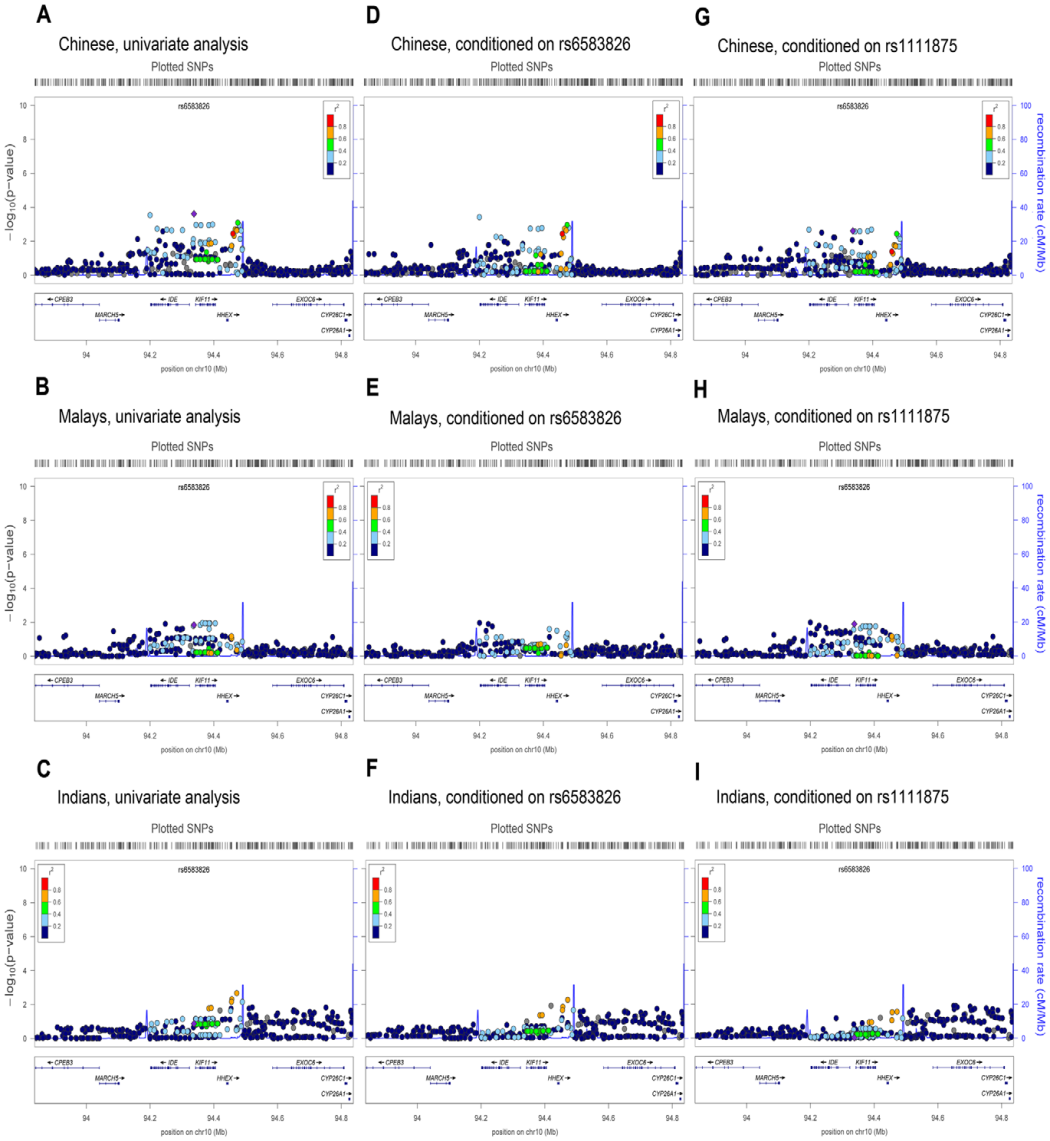
Regional association plots of the index SNP in *HHEXX/IDE/KIF11*. For each ethnic group, the univariate analysis regional plot, A) Chinese B) Malays C) Indians, is shown together with analysis conditioned on the index SNP rs6583826, D) Chinese E) Malays, F) Indians, found in meta-analysis across three ethnic groups and established index SNP rs1111875, G) Chinese H) Malays I) Indians, in populations of European ancestry.

The regional associations around the *HHEX*, *IDE* and *KIF11* genes on chromosome 10 appeared to be considerably different across the three populations, with suggestive statistical evidence spanning all three genes in the Chinese (Figure 3A); marginal evidence mainly around *KIF11* in the Malays (Figure 3B); and marginal evidence around *HHEX* in the Indians (Figure 3C). The top SNP that emerged from our meta-analysis (rs6583826) is located 115kb upstream of the SNP identified in European populations (rs1111875), suggesting that either (i) this represents only the combined signal at *KIF11* across three populations and may not be related to the associations observed at *HHEX* and *IDE*; or (ii) the LD in this region is substantially different between our populations and the European populations. To investigate the first hypothesis, we performed a conditional analysis with respect to the top SNP (rs6583826) from our meta-analysis. We observed that the association signals in the Indians were attenuated significantly (Figure 3F). This was not the case in the Chinese and Malays. In particular, SNPs located in the *IDE* gene (Figure 3D–3E) were not affected by conditioning on the top SNP. This suggests that there may be different variants associated with T2D across these three loci in different ethnic groups. We next performed a formal assessment of the extent of LD variation between reference populations for Europeans and the three ethnic groups in Singapore (Materials and Methods) in a 400 kb region centered on rs1111875 (Table S4). We found evidence of LD variation between Europeans and both Singapore Chinese (varLD monte carlo *P* = 3.00×10^−4^) and Singapore Malays (*P* = 1.21×10^−2^), but not between the Europeans and Singapore Indians (*P* = 0.24). We also performed conditional analysis with respect to the European index SNP rs1111875 (Figure 3G–3I) and rs5015480 (Figure S6). There were no perceptible changes in the regional signals for the Chinese and Malays (Figure 3G and 3H), although the association signals in the Indians appeared to have attenuated, consistent with the lack of LD variation between Europeans and Indians (Figure 3I).

## Discussion

We have performed three genome-wide case-control surveys of T2D in Chinese, Malays and Asian Indians in Singapore. To the best of our knowledge, this is the first GWAS to be performed in Malays, which forms the largest population group in Southeast Asia, comprising more than 300 million people. A meta-analysis of these three studies showed strong associations at the *CDKAL1* and *HHEX*/*IDE*/*KIF11* loci that have consistently emerged in T2D studies in multiple global populations. More importantly, the availability of genome-wide data across these three major population groups allowed us to evaluate the transferability and relevance of the previously established genomic regions that are involved in the pathogenesis of T2D. In addition, we investigated the genetic architecture at *CDKAL1* and *HHEX*/*IDE*/*KIF11*, showing evidence of population-specific effects, allelic heterogeneity and LD variations at these loci across our three populations.

Of note, our study failed to detect statistically significant associations for a number of the variants that had been discovered and validated in previous studies, mostly in European populations.

One potential reason relates to the power of our studies to detect these associations. Several things contribute to the limited power in our studies. Firstly, the sample sizes in our individual studies were relatively small, especially compared to the European consortia. Secondly, at several of these loci, the allele frequencies were lower in our studies than in European populations. The impact of low allele frequencies is exemplified for variants at the *TCF7L2* locus, which exhibited the greatest effects on T2D risk in European populations. The frequencies of the risk allele at *TCF7L2* (rs7903146) were 0.023 in the Chinese and 0.043 in the Malays, compared to 0.285 in the Indians (which is similar to that observed in European populations). While the direction and magnitude of the effects sizes were similar in our populations and previous reports [24], [49]–[50], statistical significance was only observed in the Indian GWAS. This impact of low allele frequency is consistent with the finding in a Japanese population that found associations with TCF7L2 variants with T2D in the same direction in European populations, when the sample size was sufficiently large [20]. Based on the allele frequencies observed in our studies and the sample sizes available, we had at least 80% power to detect the associations for only 8 (*TCF7L2*, *HNF1B*, *UBE2E2*, *CDKAL1*, *SLC30A8*, *HHEX*, *KCNQ1* and *SRR*) of the 36 variants in the Chinese (the ethnic group with the largest sample size), 1 in the Malays (*SRR*) and 2 in the Indians (*TCF7L2* and *SRR*). A third reason for the lack of power in our studies is that the effect sizes in our study were generally smaller than those observed in the initial studies that identified these variants. It is possible that the effect estimates of the initial discoveries were over-estimates (winner's curse). Other potential reasons for this include allelic heterogeneity or LD variation between populations which are discussed in the following paragraphs. For several of the variants where we had at least 80% power to detect an association at α = 0.05, the effect sizes seen in our studies were smaller than those reported in the initial European populations. These include variants at the *TCF7L2*, *SLC30A8* and *KCNQ1* loci. It is noteworthy that the variants for which we were able to detect an association in our study were mostly discovered in the earlier wave of genome-wide scans for T2D (including *CDKAL1*, *HHEX/IDE/KIF11*, *IGFBP2*, *SLC30A8* and *FTO*) [5]–[7], [51]. These early studies had smaller sample sizes and thus, variants identified generally had larger OR than those that emerged when large number of individuals were analyzed jointly (as is typical of meta-analyses of populations with European ancestry).

Despite the limited power of our study to detect statistically significant associations, our study showed that, of the variants identified in European GWAS for T2D, there were more statistically significant associations detected in our study than could be expected by chance, and that the direction of association was consistent in our study and in studies conducted in European populations. This suggests that for many of the variants identified in European populations, the findings are likely to be relevant in Asian populations.

The second reason for the failure to detect these associations may be due to the presence of allelic heterogeneity, where different and/or multiple causal variants may be responsible for the association signals observed at a locus in different populations. There is suggestive evidence from our study that *CDKAL1* may harbour at least two separate functional polymorphisms in the Indians where adjustment for the lead SNP attenuated a set of signals but boosted the evidence in an upstream intronic region. Similarly, at the *HHEX*/*IDE*/*KIF11* locus, there is evidence to suggest that the associations seen in Chinese (and to a certain extent, in Malays) originate from at least two separate functional polymorphisms, where the lead SNPs from our study (rs6583826) and from European studies (rs1111875) do not entirely account for the association signals observed at this locus. This finding is consistent with evidence that recently emerged from a large meta-analysis in populations of European ancestry that also identified more than 1 independent signal at several loci that were associated with T2D [11].

Finally, even when the same causal variant at a locus is present in different populations, heterogeneous patterns of LD between the causal variant and the genotyped SNPs may result in different variants emerging from GWAS in these populations. This can critically confound meta-analyses, which fundamentally assume that the same index SNP is implicated across multiple populations. Our analyses using varLD show that, for most T2D associated loci, significant LD variation exists between all pairs of ethnic groups, except Chinese and Malays. Unfortunately, despite this evidence of LD variation, our power calculations suggest that our studies are not adequately powered to detect these associations in the individual ethnic groups. As such, we are not able to examine heterogeneity between ethnic groups for most of these loci (apart from *CDKAL1* and *HHEX/IDE/KIF11*). In such settings, statistical methods for assessing regional evidence across multiple studies without relying on observing phenotype associations at the same index SNP may be increasingly relevant and important.

One important caveat of our study is the age profile of our control samples, which are generally younger than the cases, especially in the Chinese cohort. As some of the control individuals may subsequently develop T2D, the use of young controls likely has the effect of reducing statistical power and under-estimating the effect sizes. However, the signals of association at *CDKAL1* are useful for calibrating the extent of this, and the effect sizes in our Chinese and Indian populations were similar or even larger than those reported in European studies despite comparable frequencies of the implicated alleles.

The advent of GWAS allows an unbiased survey to be made across the entire human genome for novel genetic loci that are causally responsible for T2D onset. Presently, it is difficult to assess the implications of these genetic discoveries to global public health, primarily because these findings are typically identifying surrogate markers that likely do not have any bearing on genetic etiology of T2D. Fine-mapping the polymorphisms that are biologically responsible will present a significant advancement in addressing the relevance of any genetic discoveries in other populations. However, genetic and/or environmental modifiers resulting in population-specific effects and allelic heterogeneity can continue to confound the situation, even when the causal variants have been identified. This may have implications for subsequent studies that attempt to uncover the genetic architecture of T2D. It highlights the importance of conducting genetic surveys in T2D across multiple populations, particularly those that are likely to experience the greatest increase in T2D burden. This is even more pertinent as interest in medical genetics gradually shifts towards searching for rare variants, which are exactly those that are even more likely to be exclusive to specific populations.

## Materials and Methods

### Ethics statement

Ethics approval has been granted for the sample recruitment by the Singapore General Hospital Ethics Committee and the Singapore Eye Research Institute Ethics Committee. In addition, the genetic analysis was approved by the National University of Singapore Institutional Review Board (Approval Certificate NUS465).

### Study populations

The Singapore Diabetes Cohort Study (SDCS) is a research initiative led by the National University of Singapore together with the National Healthcare Group Polyclinics, National University Hospital Singapore and Tan Tock Seng Hospital. Its primary aim is to identify genetic and environmental risk factors for diabetic complications especially diabetic nephropathy, and to develop novel biomarkers for tracking disease progression. Since 2004, all type 2 diabetes patients seen at the polyclinics and hospitals were invited to be part of the cohort. Questionnaire data as well as clinical data of consenting patients were obtained together with bio-specimens such as blood and urine archived at −80°C. The participation response rate is excellent at more than 90% and to date, there are more than 5,000 patients in SDCS. For the purpose of this study, 2,202 Chinese subjects were available for genome wide analysis.

The Singapore Prospective Study Program (SP2) includes 6,968 participants from one of four previous cross-sectional studies: Thyroid and Heart Study 1982–1984 [52], National Health Survey 1992 [53], National University of Singapore Heart Study 1993–1995 [54] or National Health Survey 1998 [55]. All studies involved a random sample of individuals from the Singapore population, aged 24 to 95 years, with disproportionate sampling stratified by ethnicity to increase the number of minority ethnic groups (Malays and Asian Indians). From 2003–2007, 10,747 participants were invited to participate by linking their unique national identification numbers with national registries, where 7,742 attended the interview and of these 7,742 participants, 5,163 attended the clinical examination. Detailed population selection and methodology have been previously reported. A total of 5,499 Chinese, 1,405 Malays and 1,138 Asian-Indians were available at the time of the study and only the Chinese were used for this study.

The Singapore Malay Eye Study (SiMES) is a population-based, cross-sectional study of Malay adults (N = 3,280), aged 40 – 80 years living in Singapore. Of the 4,168 eligible participants invited, 3,280 participated in the study with a 78.7% response rate. Briefly, age-stratified random sampling of all Malay adults aged from 40–80 years residing in 15 residential districts in the southwestern part of Singapore was performed. Details of the study participants and methods have been published previously [56].

The Singapore Indian Eye Study (SINDI) is a population-based, cross-sectional study of Asian Indian adults (N = 3,400), aged 40–80+ years residing in the South-Western part of Singapore, as part of the Singapore Indian Chinese Cohort Eye Study. Age stratified random sampling was used to select 6,350 eligible participants, of which 3,400 participated in the study (75.6% response rate). Detailed methodology has been published [57].

Chinese cases included individuals with diagnosis of T2D from SDCS while Chinese controls were individuals with no prior history of diabetes and had a fasting glucose level of not more than 6.0 mmol/L selected from SP2, giving a total of 2,010 cases from SDCS and 1,945 controls from SP2 post genotype QC. In both SiMES and SINDI, cases and controls were selected from the population based cross sectional studies where diabetic cases were defined as having either a history of diabetes or had HbA1c level greater than or equal to 6.5%. Controls had no history of diabetes and HbA1c level less than 6% [58]. This yielded 794 Malay diabetic cases with 1,240 controls and 977 Indian diabetic cases with 1,169 controls.

### Genotyping

4,693 blood-derived samples, 2,210 cases from SDCS and 2,483 controls from SP2 study, were genotyped using Illumina BeadStation, Illumina HumanHap 610 Quad, and 1Mduov3 Beadchips (http://www.illumina.com/), with 16 samples (8 random cases and 8 random controls) genotyped on both Beadchips. The mean SNP concordance rate of 99.9% between chips for the post-QC duplicated samples was computed based on 531,805 post-QC common SNPs between chips. 2,662 samples were genotyped on the 610Quad and 2,031 samples on the 1Mduov3. For each array in each cohort, a first round of clustering was performed with the proprietary clustering files from Illumina (GenCall). Samples achieving a 99% call rate were subsequently used to generate local clusterfiles (GenTrain) which were used for a final round of genotype calling. A threshold of 0.15 was implemented on the GenCall score to decide on the confidence of the assigned genotypes.

For the SiMES study, 3,072 samples from the population based study were genotyped on the Illumina HumanHap 610Quad. The same procedure of genotype calling used for the Chinese was implemented in the Malays.

For the SINDI study, 2,953 samples were genotyped on the Illumina HumanHap 610Quad and identical genotype calling procedures were applied.

### Quality control (QC)

For each genotyping chip in individual cohorts, QC criteria included a first round of SNP QC to obtain a pseudo-cleaned set of genotypes for sample QC. SNPs that had missingness >5% or gross departure from HWE (*P*<10^−6^) or were monomorphic were temporarily removed from the data. Samples were then removed based on the following conditions: sample missingness, excessive heterozygosity, cryptic relatedness, discordant ethnic membership and gender discrepancy. Bivariate plots of sample call rates and heterozygosity, defined as the proportion of heterozygous calls of all valid autosomal genotypes in an individual, are used to assess the overall distribution of missingness and heterozygosity across all the samples. Cryptic relatedness by IBS computation for all pairwise combinations of samples identified first degree relatives such as monozygotic twins/duplicates, parent-offspring pairs and full-sibling pairs and only one sample from each relationship will be retained for further analysis. Samples with gender discrepancies between the genetically inferred gender from Beadstudio and clinical reported gender were removed. Population structure ascertainment was carried out using principal components analysis (PCA) [59] with 4 panels from International Hapmap [60] and the Singapore Genome Variation Project [61] (http://www.nus-cme.org.sg/SGVP/) which includes 96 Chinese, 89 Malays and 83 Asian Indians from Singapore. We used a thinned set of SNPs evenly spaced across the genome to reduce LD. The PCA plots are shown in Figure S2. Individuals who showed discordant ethnic membership from their self-reported ethnicity were excluded from the analysis. For the Malays and Indians which showed a continuous cloud suggesting some degree of admixture, the principal components were useful for correction of population structure in association testing (Figure S2F–S2H). A final round of SNP QC was then applied, removing SNPs that had missingness >5% or gross departure from HWE (p-value<10^−4^) or were monomorphic. Minor allele frequency threshold is not used. We visually assess clusterplots for every SNP with an association p-value<10^−4^ in either the individual GWAS or the meta-analysis.

The above procedure led to the exclusion of 296 samples genotyped on the 610Quad and 141 samples genotyped on the 1Mduov3 for the Chinese cohort. Samples were then checked for cryptic relatedness and gender discrepancies across the two chips resulting in another 139 samples removed. Among 542,201 post-QC SNPs on the 610Quad samples and 944,144 post-QC SNPs on the 1Mduov3 samples, common SNPs across the chips were also checked for differences in allelic differences separately in the cases and controls. 97 SNPs showing significant deviation from the null of no difference in allele frequencies were removed. Lastly, controls with fasting glucose >6 mmol/L were excluded from the analysis. In summary, the post-QC dataset consists of 1,082 cases and 1,006 controls genotyped on the 610Quad and 928 cases and 939 controls on the 1Mduov3. For the Malay study population, 530 samples were excluded due to sample call rates, cryptic relatedness, issue with population structure ascertainment and gender discrepancies. In all, 557,824 SNPs for 2,542 samples were available for analysis after applying the above quality filter procedures. For the Indian study population, 415 samples were excluded due to sample call rates, cryptic relatedness, issue with population structure ascertainment and gender discrepancies. Finally, 559,119 SNPs in 2,538 samples were available for analysis after applying the above quality filter procedures (Table S5).

### Imputation

The final post-QC set of genotype data was used to impute SNPs on the International HapMap Phase 2 panels using IMPUTE v0.5.0 [62] (https://mathgen.stats.ox.ac.uk/impute/impute.html). Imputation was carried out using the JPT+CHB panel on build 36 release 22 for the Chinese, while all 4 panels of Hapmap II were combined as a mixture reference set of the Malays and Indians. The genotype data were split into chunks of 10 Mb and imputed with a buffer of 250 kb was implemented to avoid edge effects. The effective population size of the YRI panel (Ne = 17469) was used when imputing against the combined reference panel. In addition, the known T2D regions (Table S3) were imputed with IMPUTE v2.1.2 [63], incorporating population-specific genotypes from SGVP. Specifically, for imputing the Chinese samples, we have used the HapMap JPT+CHB haplotypes as the base reference and the SGVP Chinese genotypes as additional unphased reference. For imputing the Malays, we have used the phased haplotypes from all three population panels in HapMap2 as base reference, and the SGVP Malay genotypes as additional unphased reference. For the Indian samples, we have similarly used all the phased haplotypes in HapMap2, with the addition of SGVP Indian genotypes as additional unphased reference. A 5 Mb region was imputed around the lead SNP with similar buffer size and effective population size as the genome-wide imputation. Thresholded call rates and the information score were used as the imputation metrics. For association testing, we only included imputed SNPs that had a call rate of 95% when thresholded at 0.90 posterior probability and proper_info >0.5.

### Association tests

Logistic regression using the additive mode of inheritance was performed to test the association between type 2 diabetes and the SNPs on SNPTESTv1.1.5 [51] (http://www.stats.ox.ac.uk/~marchini/software/gwas/snptest.html). Chromosome X was not tested for association in each of the case control studies. The first two principal components from PCA were used as covariates in the association tests for the Malays to adjust for population admixture while three principal components were required for the Indians (Figure S2). Genotype imputation uncertainties were incorporated in the association analyses with imputed data, using the -proper option in SNPTEST. For SNPs typed on the genotyping arrays, the experimentally-determined genotypes are reported and imputed results are not used in association testing. For the Chinese cohort, the association tests were carried out by treating the samples from separate chips as independent studies and the fixed-effects inverse-variance method of meta-analysis was used to obtain an overall association result for the Chinese. We have previously shown that SNPs associated with T2D in European populations also show similar associations in populations of Asian ethnicity [24]. As such, to improve the power of our study for discovery, the results from the combined Chinese analysis were meta-analysed together with the Malays and Indians using fixed effects inverse-variance modelling using METAL (http://www.sph.umich.edu/csg/abecasis/Metal/index.html). The complete process from QC to association testing is depicted in Figure S1. For each case control study, the manhattan plots of the association *P*-values and bivariate plots of observed –log_10_(*P*-values) against the expected –log_10_(*P*-values) are shown in Figure S3. In addition, the genotype clustering of observed genotypes with statistical significance<10^−4^ in the meta-analysis and top 10 regions of the individual GWAS are visually assessed, and SNPs with indication of ambiguous genotype calls are subsequently removed from the analyses. Genomic control was applied to the individual cohorts and to the combined meta-analysis, by inflating the standard error of the log-odds with the genomic inflation factors. A *P*-value cutoff of 5×10^−8^ was used to declare genome-wide significance for any novel polymorphism associated with T2D.

### Comparing effect of risk alleles with SNPs reported in T2D studies of European descent

The direction of effect for each SNP in each ethnic group and combined meta-analysis were compared with those derived from populations of European descent in established T2D lead SNPs. A binomial test under the null hypothesis of probability of concordance in direction of OR for the same allele at 0.5 was performed to assess whether the observed concordance was due to chance. A second binominal test was performed to investigate if the number of observed nominally significant associated loci would be expected by chance, under the null of *P* = 0.05. Lastly, in comparing the effect sizes of our populations with those derived from the European genome wide scans, a binomial test was performed to investigate if the effect sizes observed in our populations were smaller than those observed in European populations by chance. For all the above binomial tests, the meta-analysis results are only considered when information was available for all four case control studies.

### Conditional analysis

Conditional analysis was performed at *CDKAL1* and *HHEX/IDE/KIF11* in two ways, (i) by including the genotypes of the index SNPs as additional covariates to explore additional diabetes-associated SNPs in the region and (ii) by including the genotypes of the lead SNP that emerged out of T2D studies in populations of European descent (rs7754840 for *CDKAL1* and rs1111875/rs5015480 for *HHEX/IDE/KIF11*) to assess the differences in the LD between our populations and the European populations. For (i), the lead observed SNPs with directly observed genotyped, rs6583826, were used as covariates in the conditional analysis for *HHEX/IDE/KIF11*.

### Assessing linkage disequilibrium variation at the known diabetes implicated loci

The varLD algorithm [64]–[65] was used to assess regional patterns of LD variation between two populations. We considered a 400kb region centred on each index SNP and used the targeted varLD approach to evaluate the statistical significance that the pattern of correlation between every pair of SNPs in this region is similar between two populations. Briefly, a symmetric matrix of the signed r^2^ was calculated between all possible pairs of the SNPs in the region. The extent of LD difference is then given by the difference in the trace of the eigen-decomposition of the signed r^2^ matrix in the two populations. We then generated a Monte Carlo *P*-value by resampling from data combined across the two populations under the null of no differences in regional LD. We implemented 10,000 iterations for the Monte Carlo procedure. We compared the European panel (CEU) from Phase 2 of the International HapMap Project [60] with each of the three populations from the Singapore Genome Variation Project [61].

## Supporting Information

Figure S1 Flowchart summarising the study design and analysis procedures for each of the three ethnic groups.
(0.42 MB TIF)

Figure S2 Principal components analysis (PCA) plots of genetic diversity for each of the case control study and when superimposed against the Singapore Genome Variation Project (SGVP) populations. Each figure represents the genetic diversity across each ethnic group, with each individual mapped onto a spectrum of genetic variation represented by the first and second eigenvectors of the PCA. Individuals from each SGVP population is represented by a unique colour (Chinese CHS in red, Malays MAS in green and Indians INS in blue) with cases and controls for each ethnic group represented by grey and pink respectively. (A) Chinese Type 2 Diabetes (T2D) case controls with SGVP; (B) Malay T2D case controls with SGVP; (C and D) Indian T2D case controls with SGVP and (E) Chinese T2D case controls, showing first two components. No correction for population structure; (F) Malay T2D case controls, showing first two principal components used for population structure correction; (G and H) Indian T2D case controls, showing first to third components. The first three principal components were used for population structure correction.
(1.81 MB TIF)

Figure S3 Pairs of Manhattan and PP-plots of genome-wide association with T2D for each case control study separately and combined meta-analysis. Grey line on the PP-plots denotes the line y = x and the upper 95% confidence interval. (A) Chinese genotyped on the Illumina610 array (B) Chinese genotyped on the Illumina1M array (C) Chinese combined on meta-analysis (D) Malays genotyped on the Illumina610 array (E) Indians genotyped on the Illumina610 array and (F) Combined meta-analysis for all case control studies.
(1.42 MB TIF)

Figure S4 Power curves estimated for each ethnic group, based on the sample sizes in the studies for odds ratios ranging from 1.0 to 1.5 in steps of 0.01 with allele frequencies of 0.05 and 0.10 in increments of 0.05.
(0.52 MB TIF)

Figure S5 Regional plots of novel loci from meta-analysis showing *P*-values<1×10^−5^ at lead SNP, with buffer of 500kb upstream and downstream of the index SNP, which are genotyped in all the ethnic groups: (A) Chromosome 15, spanning genes *HMG20A* and *TSPAN3* (B) Chromosome 3 near *ZPLD1* (C) Chromosome 21 on the gene *HUNK* and (D) Chromosome 6 near hypothetical protein *C6orf57*.
(0.92 MB TIF)

Figure S6 Regional association plots of the index SNP in *HHEX/IDE/KIF11* in each ethnic group. For each ethnic group, the conditional analysis on the index SNP rs5015480 found in DIAGRAM+ are shown in populations our populations. (A) Chinese (B) Malays (C) Indians.
(0.68 MB TIF)

Table S1 Statistical evidence of the top regions (defined as *P*-value<10^−5^) that emerged from the single-population GWAS for Chinese, Malays and Asian Indians. For each region, the index SNP with the strongest statistical evidence is reported along with the number of SNPs within 500kb exhibiting evidence of *P*-value<10^−4^. Genomic control (GC) inflation factors for each population are also reported.
(0.06 MB DOC)

Table S2 Statistical evidence of the top regions (defined as *P*-value<10^−5^) that emerged from the fixed-effects meta-analysis of the GWAS results across Chinese, Malays and Asian Indians presented for each ethnic group.
(0.05 MB DOC)

Table S3 Known Type 2 Diabetes susceptibility loci tested for replication in the three Singapore populations separately and combined meta-analysis. Published ORs are obtained from European populations and correspond to the established ORs in Figure 2. Risk alleles are in accordance with previously established risk alleles and with information on whether each SNP is a directly observed genotype (1) or is imputed (0) or (.) is not available for analysis. Power (%) refers to the power of the individual studies to detect the published ORs at an α-level 0.05, given the allele frequency and sample sizes observed in our own studies.
(0.14 MB DOC)

Table S4 Monte-Carlo *P*-values from varLD algorithm for the 36 established T2D susceptibility loci, comparing the European panel of Hapmap II (CEU) with Chinese (CHS), Malays (MAS) and Asian Indians (MAS) in Singapore and within the three ethnic groups.
(0.11 MB DOC)

Table S5 Number of samples excluded during quality control and their reasons for exclusion. Note that the same sample may be excluded for more than one reason and each sample falls into exactly one of the exclusion reasons.
(0.05 MB DOC)

Text S1 Description of results from the individual genome wide association studies (GWAS) and meta-analysis.
(0.03 MB DOC)
