# Supplementary figures and images for: Transferability of Type 2 Diabetes Implicated Loci in Multi-Ethnic Cohorts from Southeast Asia
Source: PLoS Genet. 2011 Apr 7;7(4):e1001363. doi: 10.1371/journal.pgen.1001363 (PMC3072366; doi:10.1371/journal.pgen.1001363)

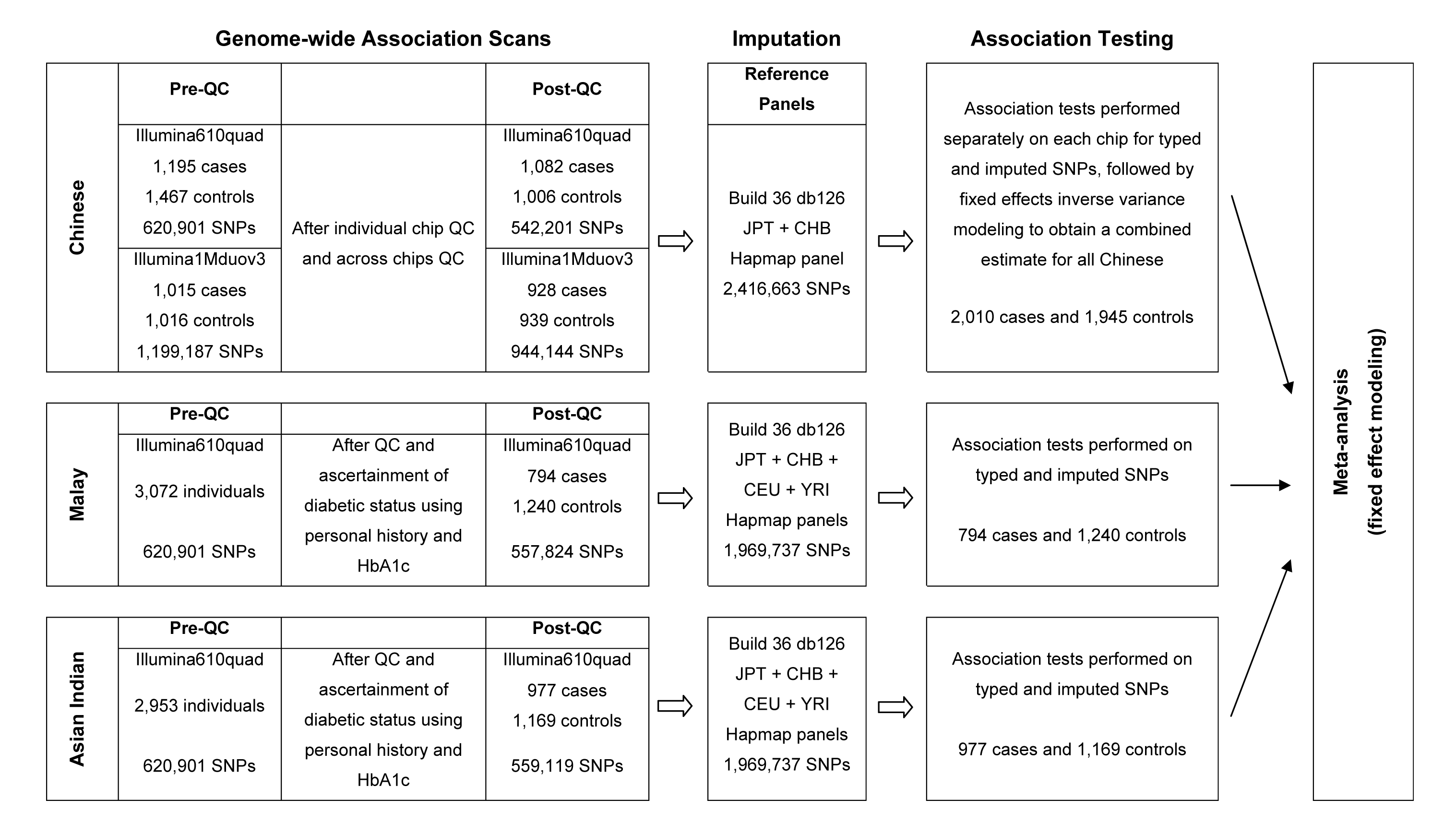

Supplement: Figure S1 — Flowchart summarising the study design and analysis procedures for each of the three ethnic groups. (0.42 MB TIF) [file pgen.1001363.s001.tif]

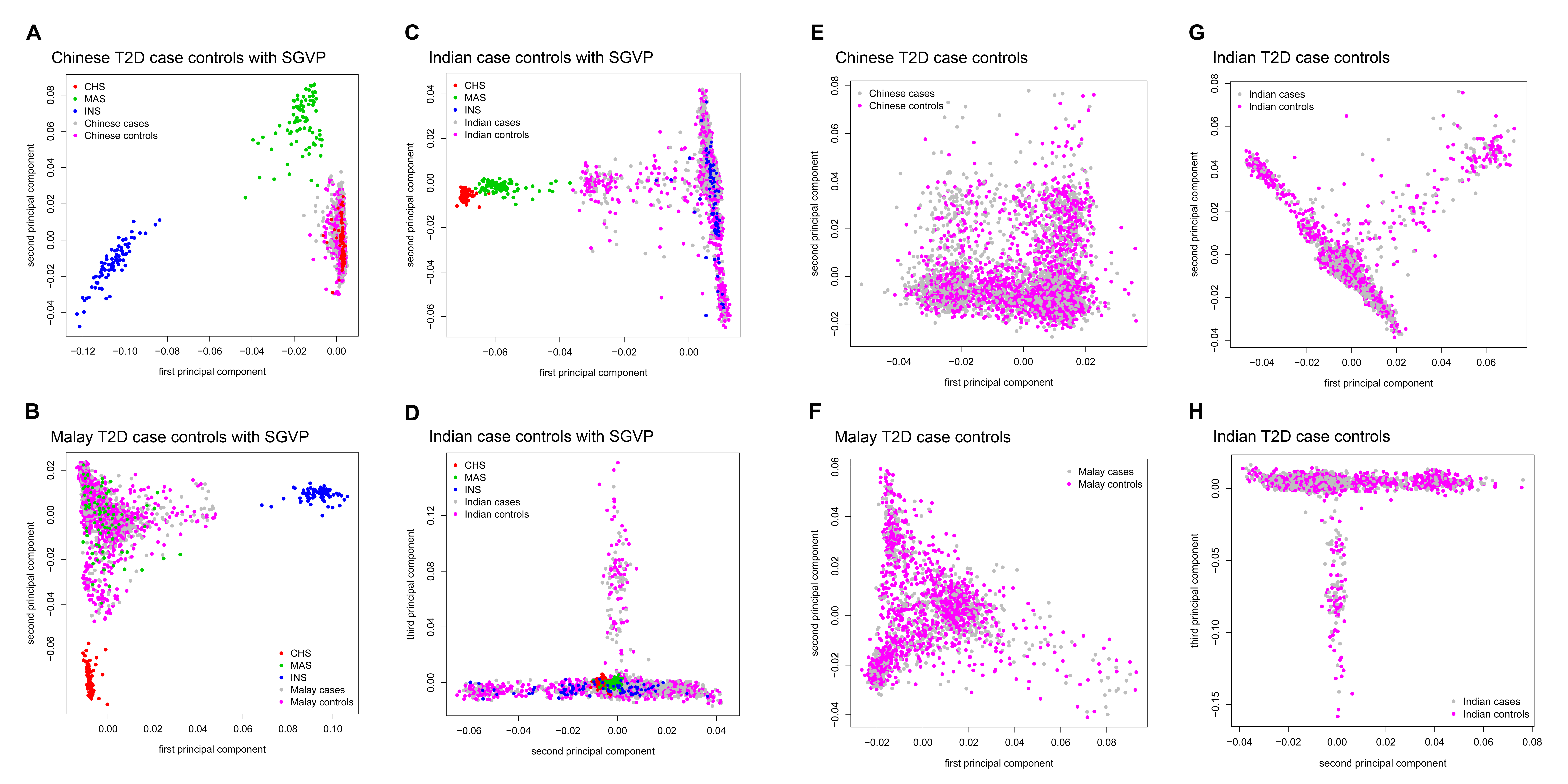

Supplement: Figure S2 — Principal components analysis (PCA) plots of genetic diversity for each of the case control study and when superimposed against the Singapore Genome Variation Project (SGVP) populations. Each figure represents the genetic diversity across each ethnic group, with each individual mapped onto a spectrum of genetic variation represented by the first and second eigenvectors of the PCA. Individuals from each SGVP population is represented by a unique colour (Chinese CHS in red, Malays MAS in green and Indians INS in blue) with cases and controls for each ethnic group represented by grey and pink respectively. (A) Chinese Type 2 Diabetes (T2D) case controls with SGVP; (B) Malay T2D case controls with SGVP; (C and D) Indian T2D case controls with SGVP and (E) Chinese T2D case controls, showing first two components. No correction for population structure; (F) Malay T2D case controls, showing first two principal components used for population structure correction; (G and H) Indian T2D case controls, showing first to third components. The first three principal components were used for population structure correction. (1.81 MB TIF) [file pgen.1001363.s002.tif]

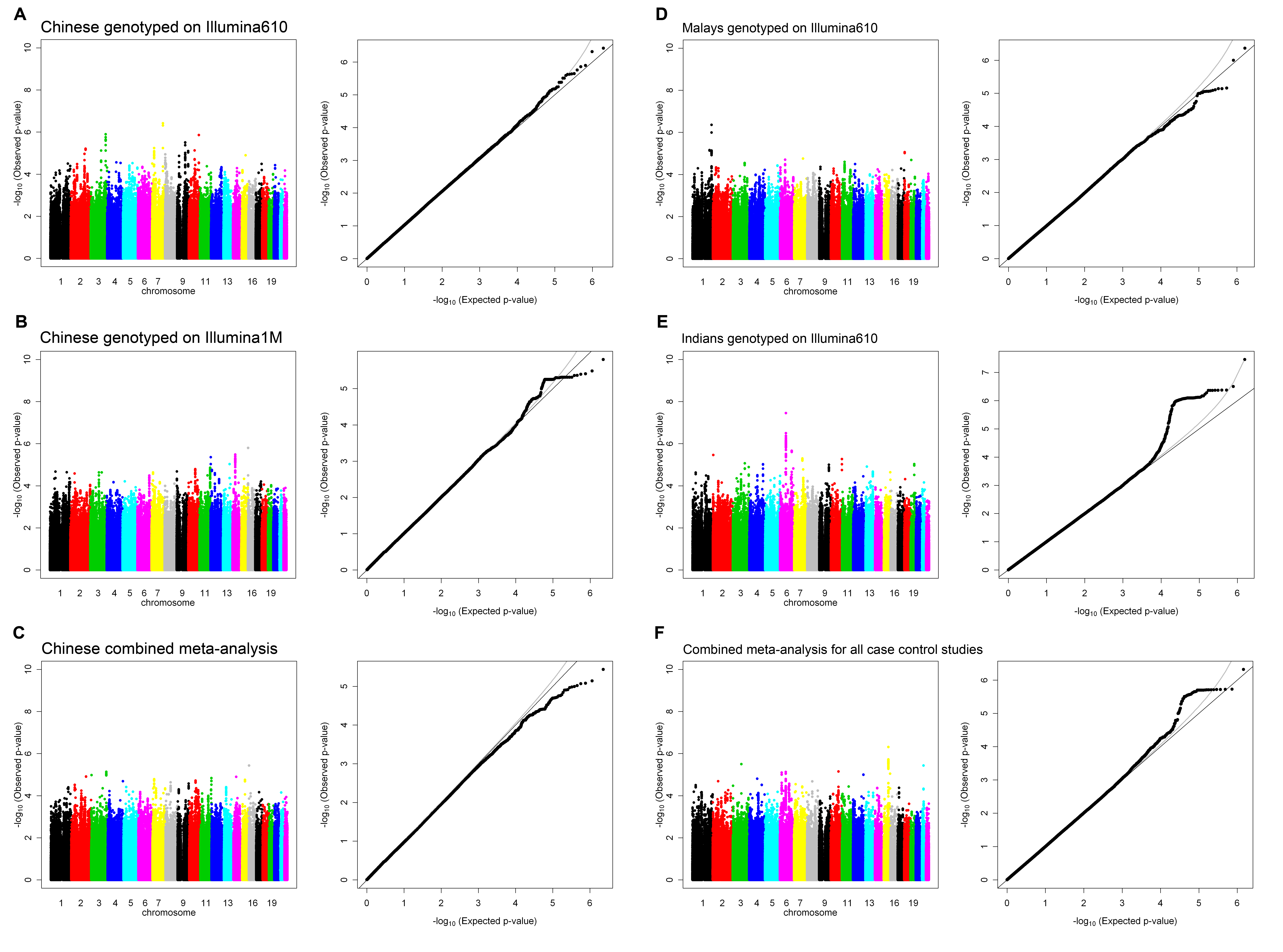

Supplement: Figure S3 — Pairs of Manhattan and PP-plots of genome-wide association with T2D for each case control study separately and combined meta-analysis. Grey line on the PP-plots denotes the line y = x and the upper 95% confidence interval. (A) Chinese genotyped on the Illumina610 array (B) Chinese genotyped on the Illumina1M array (C) Chinese combined on meta-analysis (D) Malays genotyped on the Illumina610 array (E) Indians genotyped on the Illumina610 array and (F) Combined meta-analysis for all case control studies. (1.42 MB TIF) [file pgen.1001363.s003.tif]

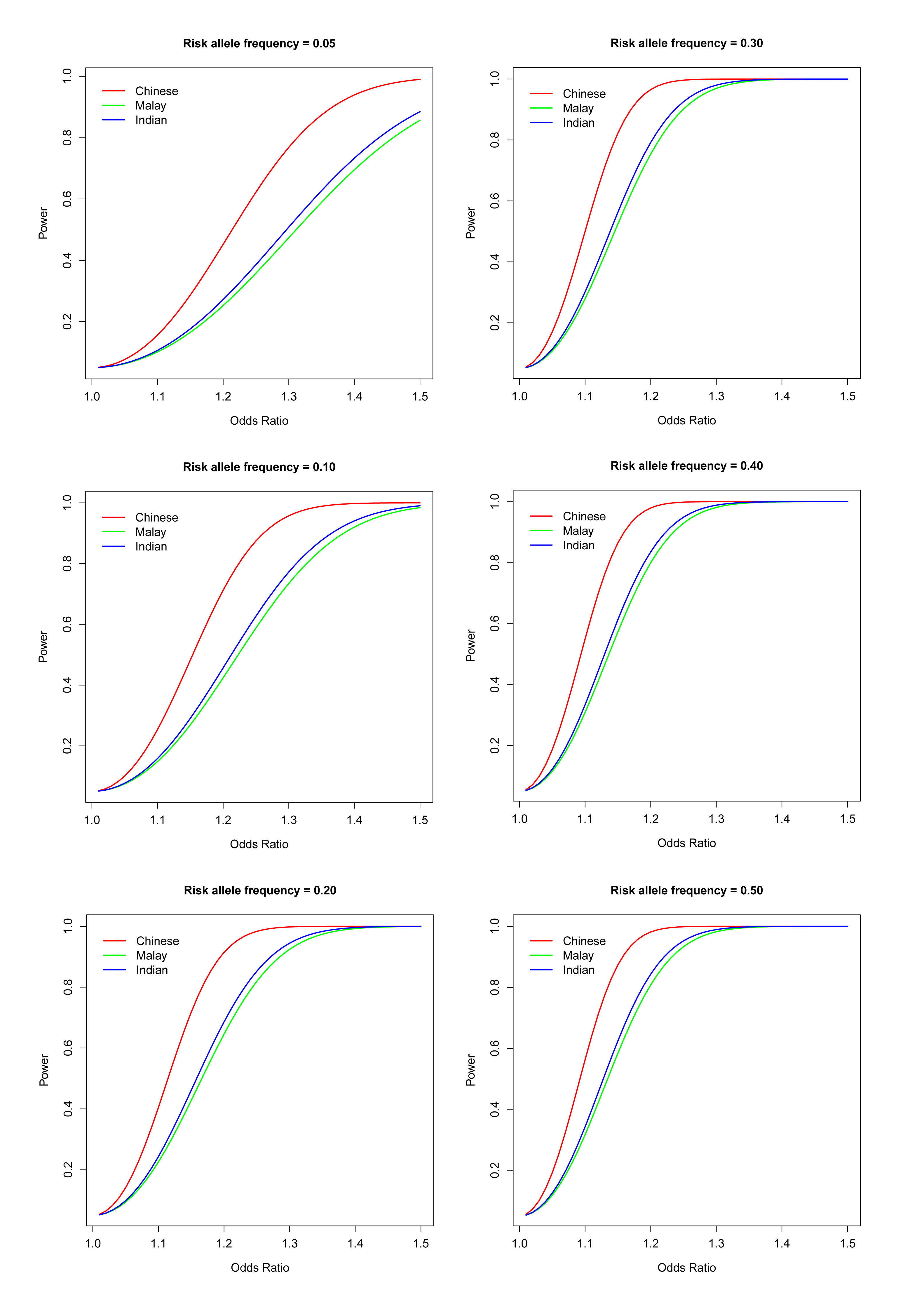

Supplement: Figure S4 — Power curves estimated for each ethnic group, based on the sample sizes in the studies for odds ratios ranging from 1.0 to 1.5 in steps of 0.01 with allele frequencies of 0.05 and 0.10 in increments of 0.05. (0.52 MB TIF) [file pgen.1001363.s004.tif]

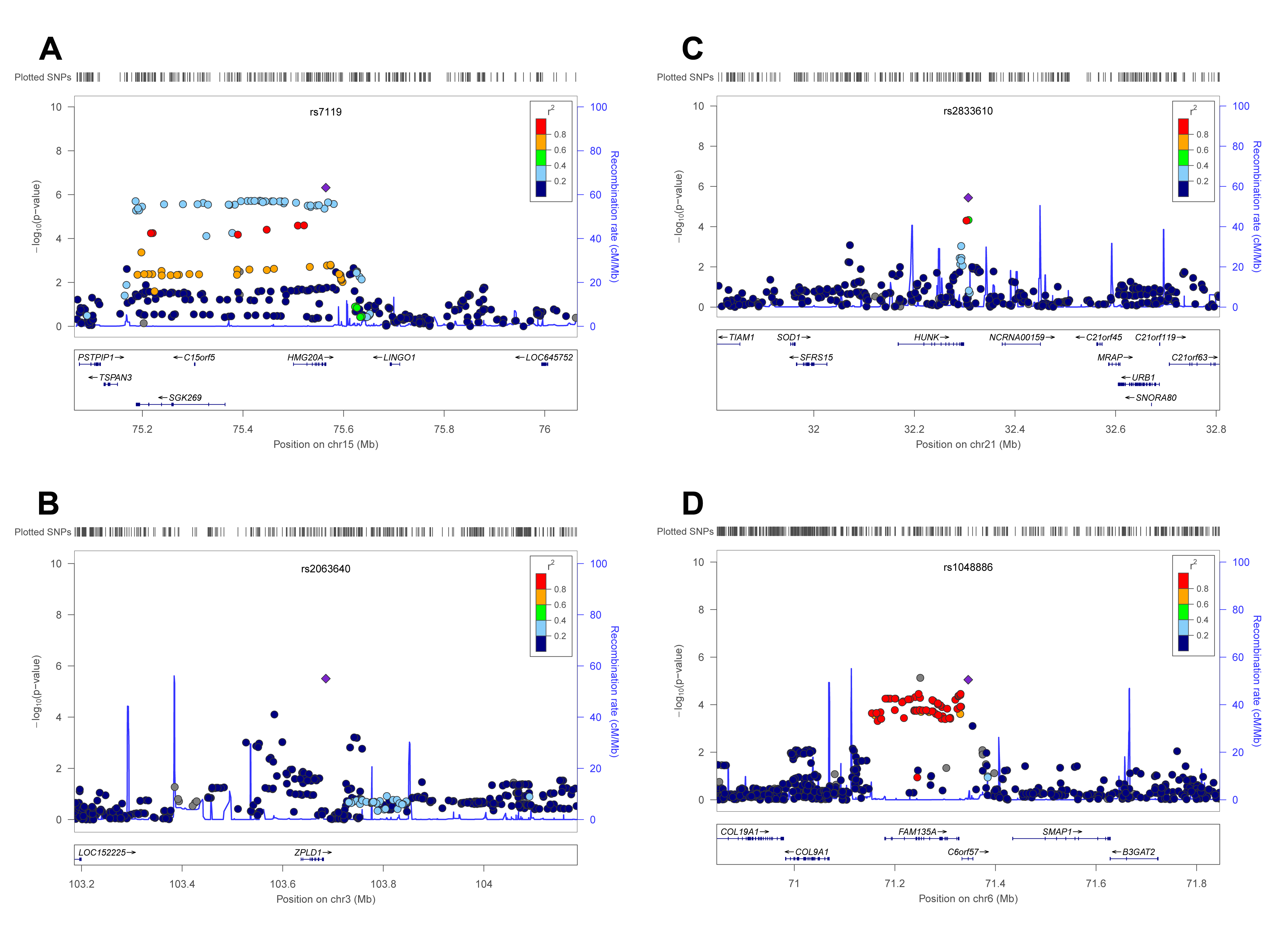

Supplement: Figure S5 — Regional plots of novel loci from meta-analysis showing P-values<1×10^−5^ at lead SNP, with buffer of 500kb upstream and downstream of the index SNP, which are genotyped in all the ethnic groups: (A) Chromosome 15, spanning genes HMG20A and TSPAN3 (B) Chromosome 3 near ZPLD1 (C) Chromosome 21 on the gene HUNK and (D) Chromosome 6 near hypothetical protein C6orf57. (0.92 MB TIF) [file pgen.1001363.s005.tif]

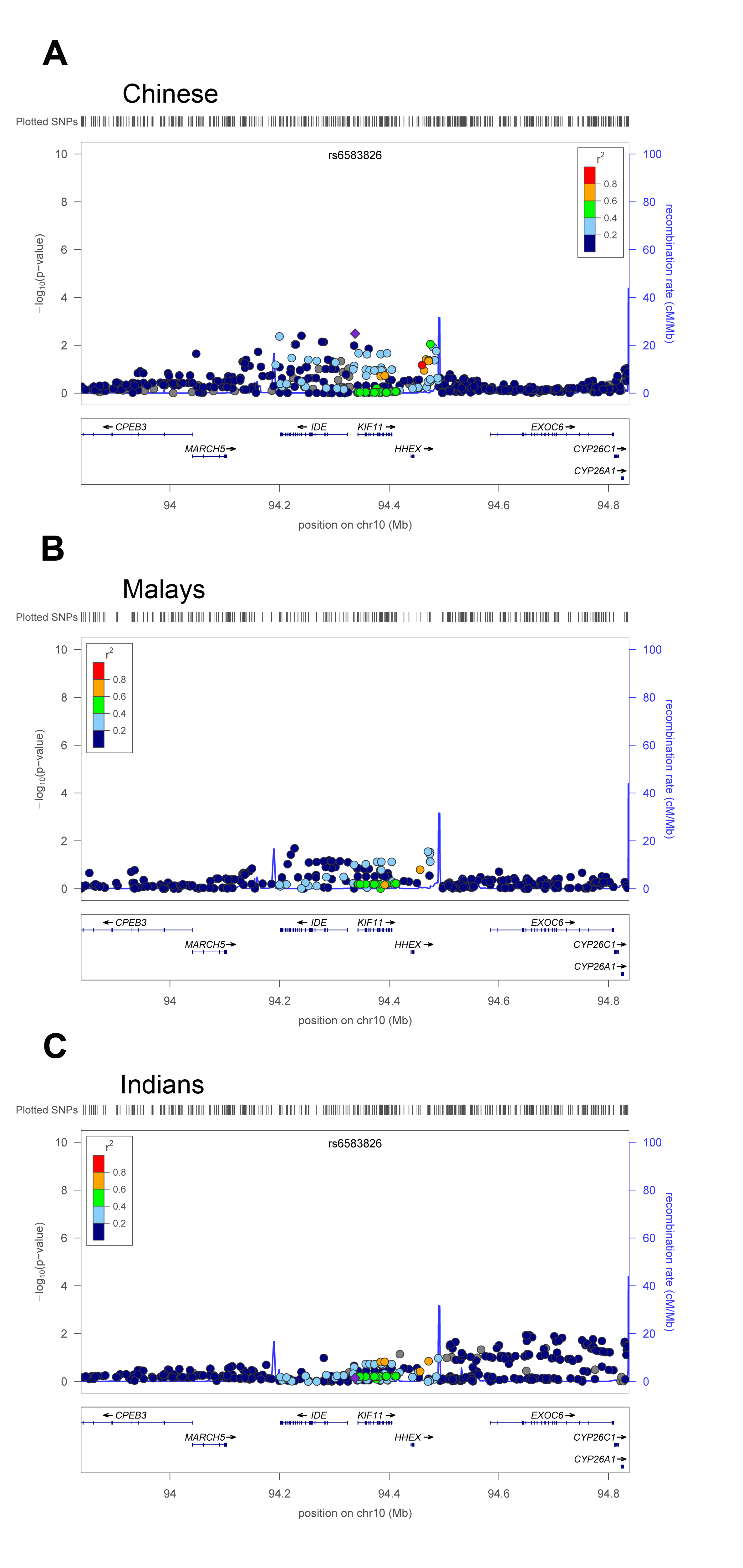

Supplement: Figure S6 — Regional association plots of the index SNP in HHEX/IDE/KIF11 in each ethnic group. For each ethnic group, the conditional analysis on the index SNP rs5015480 found in DIAGRAM+ are shown in populations our populations. (A) Chinese (B) Malays (C) Indians. (0.68 MB TIF) [file pgen.1001363.s006.tif]
