# Supplementary material for: Transferability of Type 2 Diabetes Implicated Loci in Multi-Ethnic Cohorts from Southeast Asia
Source: PLoS Genet. 2011 Apr 7;7(4):e1001363. doi: 10.1371/journal.pgen.1001363 (PMC3072366; doi:10.1371/journal.pgen.1001363)
Supplement: Table S1 — Statistical evidence of the top regions (defined as P-value<10−5) that emerged from the single-population GWAS for Chinese, Malays and Asian Indians. For each region, the index SNP with the strongest statistical evidence is reported along with the number of SNPs within 500kb exhibiting evidence of P-value<10−4. Genomic control (GC) inflation factors for each population are also reported. (0.06 MB DOC) [file pgen.1001363.s007.doc]

| **SNP** | **Chr** | **Pos (bp)** | **Risk**  **allele** | **Reference**  **allele** | **Nearest gene** | **Risk allele frequency** | **OR (95%CI)** | ***P*** | **# SNPs with *P*-value < 10^-4^** |
| --- | --- | --- | --- | --- | --- | --- | --- | --- | --- |
| **Chinese GWAS, GC = 1.017 (2,010 cases/1,945 controls)** | | | | | | | | | |
| rs7630877 | 3 | 181144012 | A | G | *PEX5L* | 0.174 | 1.32 (1.17-1.49) | 7.25 × 10^-6^ | 6 |
| **Malay GWAS, GC = 1.035 (794 cases/1,240 controls)** | | | | | | | | | |
| rs12027542 | 1 | 231406777 | A | G | *PCNXL2* | 0.614 | 1.41 (1.23-1.61) | 4.33 × 10^-7^ | 20 |
| rs17045328 | 1 | 205718799 | G | A | *CR2* | 0.302 | 1.38 (1.20-1.59) | 6.92× 10^-6^ | 2 |
| rs10460009 | 18 | 2938029 | C | T | *LPIN2* | 0.596 | 1.35 (1.18-1.54) | 8.69 × 10^-6^ | 2 |
| **Indian GWAS, GC = 1.030 (977 cases/1,169 controls)** | | | | | | | | | |
| rs1048886 | 6 | 71345910 | G | A | *C6orf57* | 0.184 | 1.54 (1.32-1.80) | 3.48 × 10^-8^ | 93 |
| rs642858 | 6 | 140315340 | A | G | NA | 0.403 | 1.35 (1.19-1.53) | 2.15 × 10^-6^ | 10 |
| rs11677370 | 2 | 3819295 | T | A | NA | 0.397 | 1.35 (1.19-1.53) | 3.39 × 10^-6^ | 1 |
| rs7636 | 7 | 100328013 | A | G | *ACHE* | 0.055 | 1.85 (1.42-2.41) | 4.99 × 10^-6^ | 5 |
| rs10741243 | 10 | 132837952 | G | C | *TCERG1L* | 0.934 | 1.75 (1.38-2.23) | 5.33 × 10^-6^ | 3 |
| rs3773506 | 3 | 143913690 | C | G | *PLS1* | 0.059 | 1.81 (1.39-2.35) | 8.51 × 10^-6^ | 4 |
| rs472265 | 19 | 44272577 | G | A | *FLJ16165* | 0.218 | 1.39 (1.20-1.61) | 9.19 × 10^-6^ | 3 |
| rs3792615 | 4 | 164752251 | T | C | *MARCH1* | 0.953 | 1.93 (1.45-2.59) | 9.38 × 10^-6^ | 5 |
| rs4457406 | 9 | 114933365 | T | C | NA | 0.350 | 1.35 (1.18-1.54) | 9.95 × 10^-6^ | 5 |
