# Supplementary material for: Transferability of Type 2 Diabetes Implicated Loci in Multi-Ethnic Cohorts from Southeast Asia
Source: PLoS Genet. 2011 Apr 7;7(4):e1001363. doi: 10.1371/journal.pgen.1001363 (PMC3072366; doi:10.1371/journal.pgen.1001363)
Supplement: Table S2 — Statistical evidence of the top regions (defined as P-value<10−5) that emerged from the fixed-effects meta-analysis of the GWAS results across Chinese, Malays and Asian Indians presented for each ethnic group. (0.05 MB DOC) [file pgen.1001363.s008.doc]

| **SNP** | **Chr** | **Pos (bp)** | **Nearest**  **gene** | **Risk**  **allele** | **Reference**  **allele** | **Chinese**  **(2010 cases/1945 controls)** | | | **Malays**  **(794 cases/1240 controls)** | | | **Indians**  **(977 cases/1169 controls)** | | |
| --- | --- | --- | --- | --- | --- | --- | --- | --- | --- | --- | --- | --- | --- | --- |
|  |  |  |  |  |  | **Risk**  **allele**  **freq** | **OR**  **(95% CI)** | **P-value** | **Risk**  **allele**  **freq** | **OR**  **(95% CI)** | **P-value** | **Risk**  **allele**  **freq** | **OR**  **(95% CI)** | **P-value** |
| rs7119 | 15 | 75564687 | *HMG20A* | T | C | 0.157 | 1.22 (1.07-1.38) | 2.01 x 10^-3^ | 0.157 | 1.28 (1.07-1.54) | 6.35 x 10^-3^ | 0.275 | 1.23 (1.07-1.41) | 3.34 x 10^-3^ |
| rs2063640 | 3 | 103685735 | *ZPLD1* | A | C | 0.217 | 1.24 (1.11-1.39) | 1.17 x 10^-4^ | 0.165 | 1.23 (1.04-1.46) | 1.62 x 10^-2^ | 0.077 | 1.15 (0.92-1.45) | 2.25 x 10^-1^ |
| rs2833610 | 21 | 32307057 | *HUNK* | A | G | 0.568 | 1.16 (1.05-1.27) | 2.02 x 10^-3^ | 0.612 | 1.10 (0.96-1.25) | 1.83 x 10^-1^ | 0.524 | 1.24 (1.10-1.41) | 5.14 x 10^-4^ |
| rs6583826 | 10 | 94337810 | *KIF11* | G | A | 0.242 | 1.22 (1.09-1.35) | 2.87 x 10^-4^ | 0.272 | 1.19 (1.04-1.37) | 1.48 x 10^-2^ | 0.277 | 1.11 (0.97-1.27) | 1.34 x 10^-1^ |
| rs1048886 | 6 | 71345910 | *C6orf57* | G | A | 0.078 | 1.00  (0.84-1.19) | 9.95 x 10^-1^ | 0.095 | 1.21  (0.95-1.50) | 8.23 x 10^-2^ | 0.184 | 1.54  (1.32-1.80) | 3.48 x 10^-8^ |
| rs9295474 | 6 | 20760696 | *CKDAL1* | G | C | 0.382 | 1.19  (1.09-1.31) | 2.04 x 10^-4^ | 0.384 | 1.05  (0.92-1.20) | 4.41 x 10^-1^ | 0.284 | 1.23  (1.07-1.40) | 3.40 x 10^-3^ |
