# Supplementary material for: Transferability of Type 2 Diabetes Implicated Loci in Multi-Ethnic Cohorts from Southeast Asia
Source: PLoS Genet. 2011 Apr 7;7(4):e1001363. doi: 10.1371/journal.pgen.1001363 (PMC3072366; doi:10.1371/journal.pgen.1001363)
Supplement: Table S3 — Known Type 2 Diabetes susceptibility loci tested for replication in the three Singapore populations separately and combined meta-analysis. Published ORs are obtained from European populations and correspond to the established ORs in Figure 2. Risk alleles are in accordance with previously established risk alleles and with information on whether each SNP is a directly observed genotype (1) or is imputed (0) or (.) is not available for analysis. Power (%) refers to the power of the individual studies to detect the published ORs at an α-level 0.05, given the allele frequency and sample sizes observed in our own studies. (0.14 MB DOC) [file pgen.1001363.s009.doc]

| **SNP** | **Chr** | **Pos  (bp)** | **Nearest**  **Gene** | **Risk**  **allele** | **Ref**  **allele** | **Pub-**  **lished**  **OR** | **Genotyped (1)**  **Imputed**  **(0)**  **Not**  **Available**  **(.)^a^** | **Chinese**  **(2010 cases/1945 controls)** | | | | **Malays**  **(794 cases/1240 controls)** | | | | **Indians**  **(977 cases/1169 controls)** | | | | **Chinese + Malays + Indians**  **(3781 cases/4354 controls)** | | |
| --- | --- | --- | --- | --- | --- | --- | --- | --- | --- | --- | --- | --- | --- | --- | --- | --- | --- | --- | --- | --- | --- | --- |
|  |  |  |  |  |  |  |  | **Power** | **Risk**  **allele**  **freq** | **OR**  **(95% CI)** | ***P*-value** | **Power** | **Risk**  **allele**  **freq** | **OR**  **(95% CI)** | ***P*-value** | **Power** | **Risk**  **allele**  **freq** | **OR**  **(95% CI)** | ***P*-value** | **Fixed effects**  **OR**  **(95% CI)** | **Fixed**  **effects**  ***P*-value** | ***I^2^***  ***(%)*** |
| ***Identified Through Candidate Gene Study*** | | | | | | | | | | | | | | | | | | | | | | |
| rs1801282 | 3 | 12368125 | *PPARG* | C | G | 1.148 | 01.0 | 29 | 0.964 | 1.07  (0.84-1.35) | 5.79 x 10^-01^ | -- | -- | -- | -- | 39 | 0.889 | 1.10  (0.91-1.33) | 3.22 x 10^-01^ | 1.09  (0.94-1.26) | 2.63 x 10^-01^ | -- |
| rs5215 | 11 | 17365206 | ***KCNJ11*** | C | T | 1.093 | 1111 | 47 | 0.363 | 1.10  (1.01-1.21) | **3.63 x 10^-02^** | 27 | 0.401 | 1.16  (1.02-1.32) | **2.26 x 10^-02^** | 28 | 0.351 | 1.03  (0.91-1.16) | 6.86 x 10^-01^ | 1.10  (1.03-1.17) | **5.00 x 10^-03^** | 0 |
| ***Identified Through Linkage Study*** | | | | | | | | | | | | | | | | | | | | | | |
| rs7903146 | 10 | 114748339 | ***TCF7L2*** | T | C | 1.398 | 1111 | 94 | 0.023 | 1.14  (0.84-1.53) | 4.04 x 10^-01^ | 70 | 0.043 | 1.20  (0.87-1.64) | 2.62 x 10^-01^ | 100 | 0.284 | 1.23  (1.08-1.40) | **2.10 x 10^-03^** | 1.21  (1.08-1.36) | **8.26 x 10^-04^** | 0 |
| ***Identified Through Candidate Pathway Analysis*** | | | | | | | | | | | | | | | | | | | | | | |
| rs10010131 | 4 | 6343816 | *WFS1* | G | A | 1.11 | 0100 | 30 | 0.919 | 1.03  (0.88-1.21) | 7.32 x 10^-01^ | 26 | 0.839 | 0.86  (0.72-1.02) | 8.17 x 10^-02^ | 32 | 0.767 | 1.04  (0.90-1.20) | 6.10 x 10^-01^ | 0.98  (0.90-1.07) | 6.88 x 10^-01^ | 38.42 |
| rs757210 | 17 | 33170628 | *HNF1B*  *(TCF2)* | T | C | 1.179 | 1111 | 93 | 0.261 | 1.12  (1.01-1.24) | **2.51 x 10^-02^** | 70 | 0.338 | 0.96  (0.84-1.09) | 4.99 x 10^-01^ | 71 | 0.274 | 0.94  (0.82-1.07) | 3.63 x 10^-01^ | 1.02  (0.96-1.10) | 4.80 x 10^-01^ | 65.55 |
| ***Identified Through Type 2 Diabetes GWAS*** | | | | | | | | | | | | | | | | | | | | | | |
| rs10923931 | 1 | 120319482 | *NOTCH2* | T | G | 1.138 | 0000 | 26 | 0.026 | 0.93  (0.71-1.22) | 5.85 x 10^-01^ | 24 | 0.054 | 1.12  (0.85-1.48) | 4.21 x 10^-01^ | 47 | 0.211 | 1.05  (0.91-1.22) | 4.95 x 10^-01^ | 1.04  (0.92-1.17) | 5.17 x 10^-01^ | 0 |
| rs7578597 | 2 | 43586327 | *THADA* | T | C | 1.151 | 1111 | 29 | 0.995 | 1.37  (0.71-2.61) | 3.45 x 10^-01^ | 17 | 0.984 | 0.74  (0.45-1.23) | 2.49 x 10^-01^ | 39 | 0.879 | 0.98  (0.82-1.18) | 8.27 x 10^-01^ | 0.97  (0.82-1.15) | 7.35 x 10^-01^ | 7.07 |
| rs243021 | 2 | 60438323 | ***BCL11A*** | A | G | 1.08 | 1111 | 38 | 0.669 | 1.05  (0.96-1.16) | 2.87 x 10^-01^ | 22 | 0.536 | 0.96  (0.85-1.09) | 5.76 x 10^-01^ | 24 | 0.482 | 1.13  (1.00-1.28) | 4.75 x 10^-02^ | 1.05  (0.98-1.12) | 1.38 x 10^-01^ | 36.97 |
| rs2943641 | 2 | 226801989 | ***IRS1*** | C | T | 1.087 | 1111 | 22 | 0.931 | 1.06  (0.89-1.26) | 5.29 x 10^-01^ | 16 | 0.892 | 1.18  (0.96-1.44) | 1.14 x 10^-01^ | 21 | 0.805 | 1.15  (0.99-1.34) | 6.64 x 10^-02^ | 1.13  (1.02-1.24) | **1.92 x 10^-02^** | 0 |
| rs6780569 | 3 | 23173478 | *UBE2E2* | G | A | 1.17 | 1111 | 81 | 0.817 | 1.12  (1.00-1.25) | 5.97 x 10^-02^ | 58 | 0.791 | 1.04  (0.89-1.22) | 5.93 x 10^-01^ | 66 | 0.701 | 1.05  (0.92-1.20) | 4.75 x 10^-01^ | 1.08  (1.00-1.16) | 5.63 x 10^-02^ | 0 |
| rs4607103 | 3 | 64686944 | *ADAMTS9* | C | T | 1.096 | 000. | 53 | 0.661 | 1.03  (0.94-1.13) | 5.24 x 10^-01^ | 28 | 0.709 | 0.94  (0.82-1.09) | 4.19 x 10^-01^ | -- | -- | -- | -- | 1.00  (0.93-1.08) | 9.29 x 10^-01^ | -- |
| rs1470579 | 3 | 187011774 | ***IGF2BP2*** | C | A | 1.139 | 1111 | 77 | 0.255 | 1.15  (1.04-1.28) | **5.80 x 10^-03^** | 50 | 0.329 | 0.99  (0.86-1.13) | 8.59 x 10^-01^ | 57 | 0.469 | 1.08  (0.95-1.22) | 2.26 x 10^-01^ | 1.09  (1.02-1.16) | **1.59 x 10^-02^** | 39.17 |
| rs4457053 | 5 | 76460705 | *ZBED3* | G | A | 1.08 | 00.. | 18 | 0.055 | 1.01  (0.84-1.22) | 9.31 x 10^-01^ | -- | -- | -- | -- | -- | -- | -- | -- | 1.01  (0.84-1.22) | 9.31 x 10^-01^ | -- |
| rs7754840 | 6 | 20769229 | ***CDKAL1*** | C | G | 1.185 | 1111 | 97 | 0.369 | 1.20  (1.09-1.31) | **1.03 x 10^-04^** | 76 | 0.369 | 1.06  (0.93-1.21) | 3.95 x 10^-01^ | 71 | 0.245 | 1.16  (1.01-1.34) | **3.60 x 10^-02^** | 1.15  (1.08-1.23) | **2.34 x 10^-05^** | 12.52 |
| rs864745 | 7 | 28147081 | *JAZF1* | T | C | 1.121 | 0000 | 60 | 0.785 | 1.00  (0.89-1.11) | 9.36 x 10^-01^ | 34 | 0.758 | 1.05  (0.91-1.22) | 4.93 x 10^-01^ | 37 | 0.752 | 1.10  (0.95-1.26) | 2.07 x 10^-01^ | 1.04  (0.96-1.12) | 3.41 x 10^-01^ | 0 |
| rs972283 | 7 | 130117394 | *KLF14* | G | A | 1.07 | …. | -- | -- | -- | -- | -- | -- | -- | -- | -- | -- | -- | -- | -- | -- | -- |
| rs896854 | 8 | 96029687 | *TP53INP1* | T | C | 1.06 | 1111 | 22 | 0.258 | 1.05  (0.95-1.16) | 3.42 x 10^-01^ | 13 | 0.29 | 1.10  (0.96-1.26) | 1.80 x 10^-01^ | 15 | 0.399 | 1.02  (0.90-1.15) | 7.91 x 10^-01^ | 1.05  (0.98-1.13) | 1.47 x 10^-01^ | 0 |
| rs13266634 | 8 | 118253964 | ***SLC30A8*** | C | T | 1.149 | 1111 | 87 | 0.545 | 1.08  (0.98-1.18) | 1.07 x 10^-01^ | 58 | 0.573 | 1.11  (0.97-1.25) | 1.20 x 10^-01^ | 52 | 0.767 | 1.08  (0.94-1.25) | 2.82 x 10^-01^ | 1.09  (1.02-1.16) | **1.39 x 10^-02^** | 0 |
| rs10811661 | 9 | 22124094 | *CDKN2A/B* | T | C | 1.191 | …. | -- | -- | -- | -- | -- | -- | -- | -- | -- | -- | -- | -- | -- | -- | -- |
| rs13292136 | 9 | 81141948 | *CHCHD9* | C | T | 1.11 | 0000 | 30 | 0.913 | 0.88  (0.76-1.03) | 1.25 x 10^-01^ | 17 | 0.914 | 0.91  (0.73-1.13) | 3.83 x 10^-01^ | 24 | 0.855 | 1.03  (0.86-1.22) | 7.64 x 10^-01^ | 0.94  (0.85-1.04) | 2.17 x 10^-01^ | 0 |
| rs17584499 | 9 | 8869118 | *PTPRD* | T | C | 1.57 | 1111 | -- | 0.104 | 1.00  (0.87-1.16) | 9.91 x 10^-01^ | -- | 0.227 | 1.06  (0.91-1.23) | 4.43 x 10^-01^ | -- | 0.257 | 0.95  (0.83-1.09) | 4.46 x 10^-01^ | 1.00  (0.92-1.09) | 9.78 x 10^-01^ | 0 |
| rs12779790 | 10 | 12368016 | *CDC123/*  *CAMK1D* | G | A | 1.092 | …. | -- | -- | -- | -- | -- | -- | -- | -- | -- | -- | -- | -- | -- | -- | -- |
| rs1111875 | 10 | 94452862 | ***HHEX/IDE*** | C | T | 1.172 | 1111 | 90 | 0.3 | 1.11  (1.01-1.22) | **2.79 x 10^-02^** | 65 | 0.313 | 1.08  (0.94-1.23) | 2.81 x 10^-01^ | 71 | 0.381 | 1.16  (1.02-1.31) | **2.19 x 10^-02^** | 1.12  (1.05-1.19) | **1.09 x 10^-03^** | 0 |
| rs2237892 | 11 | 2796327 | ***KCNQ1*** | C | T | 1.141 | 1111 | 80 | 0.686 | 1.01  (0.92-1.11) | 8.71 x 10^-01^ | 50 | 0.692 | 1.28  (1.12-1.47) | **3.42 x 10^-04^** | 16 | 0.976 | 1.15  (0.74-1.77) | 5.33 x 10^-01^ | 1.09  (1.01-1.18) | **2.40 x 10^-02^** | 75.43 |
| rs231362 | 11 | 2648047 | *KCNQ1* | G | A | 1.08 | 000. | 18 | 0.913 | 0.97  (0.83-1.13) | 6.86 x 10^-01^ | 14 | 0.856 | 1.07  (0.90-1.28) | 4.51 x 10^-01^ | -- | -- | -- | -- | 1.01  (0.90-1.14) | 8.47 x 10^-01^ | -- |
| rs1552224 | 11 | 72110746 | ***CENTD2*** | A | C | 1.14 | 1111 | 43 | 0.937 | 1.35  (1.12-1.63) | **1.38 x 10^-03^** | 24 | 0.923 | 1.06  (0.84-1.34) | 6.23 x 10^-01^ | 41 | 0.818 | 0.97  (0.83-1.14) | 6.97X 10^-01^ | 1.10  (0.99-1.23) | 6.89 x 10^-02^ | 72.68 |
| rs10830963 | 11 | 92348358 | *MTNR1B* | G | C | 1.129 | .1.. | 77 | 0.426 | 0.92  (0.81-1.05) | 1.98 x 10^-01^ | -- | -- | -- | -- | -- | -- | -- | -- | 0.92  (0.81-1.05) | 1.99 x 10^-01^ | -- |
| rs1531343 | 12 | 64461161 | *HMGA2* | C | G | 1.1 | 0000 | 34 | 0.104 | 1.09  (0.95-1.27) | 2.26 x 10^-01^ | 15 | 0.076 | 1.22  (0.96-1.55) | 1.07 x 10^-01^ | 24 | 0.187 | 1.03  (0.89-1.21) | 6.72X 10^-01^ | 1.09  (0.99-1.20) | 8.49 x 10^-02^ | 0 |
| rs7961581 | 12 | 69949369 | *TSPAN8/*  *LGR5* | C | T | 1.106 | 0000 | 53 | 0.222 | 0.98  (0.88-1.09) | 7.42 x 10^-01^ | 30 | 0.219 | 1.07  (0.92-1.24) | 3.93 x 10^-01^ | 37 | 0.346 | 1.01  (0.89-1.14) | 9.33 x 10^-01^ | 1.01  (0.94-1.08) | 8.20 x 10^-01^ | 0 |
| rs7957197 | 12 | 119945069 | *HNF1A* | T | A | 1.07 | …. | -- | -- | -- | -- | -- | -- | -- | -- | -- | -- | -- | -- | -- | -- | -- |
| rs11634397 | 15 | 78219277 | *ZFAND6* | G | A | 1.06 | 01.. | 12 | 0.075 | 1.11  (0.94-1.31) | 2.33 x 10^-01^ | -- | -- | -- | -- | -- | -- | -- | -- | 1.11  (0.94-1.31) | 2.33 x 10^-01^ | -- |
| rs7172432 | 15 | 60183671 | ***C2CD4A-***  ***C2CD4B*** | A | G | 1.12 | 1111 | 68 | 0.672 | 1.13  (1.03-1.24) | **1.06 x 10^-02^** | 40 | 0.677 | 1.00  (0.87-1.14) | 9.79 x 10^-01^ | 45 | 0.593 | 1.01  (0.89-1.14) | 9.08 x 10^-01^ | 1.06  (1.00-1.13) | 6.72 x 10^-02^ | 37.7 |
| rs8042680 | 15 | 89322341 | *PRC1* | A | C | 1.07 | 1111 | 10 | 0.997 | 1.12  (0.48-2.65) | 7.90 x 10^-01^ | 8 | 0.970 | 1.75  (1.21-2.53) | **3.09 x 10^-03^** | 16 | 0.766 | 1.00  (0.87-1.16) | 9.60 x 10^-01^ | 1.08  (0.95-1.23) | 2.52 x 10^-01^ | 73.39 |
| rs9939609 | 16 | 52378028 | ***FTO*** | A | T | 1.116 | 0000 | 45 | 0.132 | 1.27  (1.11-1.45) | **3.79 x 10^-04^** | 37 | 0.300 | 1.08  (0.94-1.24) | 2.57 x 10^-01^ | 43 | 0.329 | 1.09  (0.96-1.24) | 1.96 x 10^-01^ | 1.15  (1.06-1.24) | **5.06 x 10^-04^** | 43.05 |
| rs391300 | 17 | 2163008 | *SRR* | C | A | 1.28 | 1111 | 100 | 0.655 | 0.99  (0.90-1.08) | 7.68 x 10^-01^ | 97 | 0.466 | 0.99  (0.87-1.12) | 8.48 x 10^-01^ | 98 | 0.440 | 1.00  (0.89-1.13) | 9.96 x 10^-01^ | 0.99  (0.93-1.06) | 7.63 x 10^-01^ | 0 |
| rs10425678 | 19 | 38669236 | ***PEPD*** | C | T | 1.14 | 1111 | 73 | 0.211 | 0.87  (0.78-0.97) | 9.98 x 10^-03^ | 43 | 0.224 | 1.02  (0.87-1.19) | 8.05 x 10^-01^ | 54 | 0.331 | 0.93  (0.82-1.05) | 2.51 x 10^-01^ | 0.92  (0.85-0.99) | **2.32 x 10^-02^** | 30.4 |
| ^a^ This column shows whether each SNP is directly genotyped (1) or imputed (0) in each of the case control studies shown in Table 1. Each digit represents a case control study in the following order from left to right: Chinese on Illumina610, Chinese on Illumina1M, Malays on Illumina610 and Indians on Illumina610. | | | | | | | | | | | | | | | | | | | | | | |
