# Supplementary material for: Transferability of Type 2 Diabetes Implicated Loci in Multi-Ethnic Cohorts from Southeast Asia
Source: PLoS Genet. 2011 Apr 7;7(4):e1001363. doi: 10.1371/journal.pgen.1001363 (PMC3072366; doi:10.1371/journal.pgen.1001363)
Supplement: Table S4 — Monte-Carlo P-values from varLD algorithm for the 36 established T2D susceptibility loci, comparing the European panel of Hapmap II (CEU) with Chinese (CHS), Malays (MAS) and Asian Indians (MAS) in Singapore and within the three ethnic groups. (0.11 MB DOC) [file pgen.1001363.s010.doc]

| SNP | Chr | Position | Loci | Pairwise population comparison Monte Carlo *P*-values | | | | | |
| --- | --- | --- | --- | --- | --- | --- | --- | --- | --- |
|  |  |  |  | CEU_CHS | CEU_MAS | CEU_INS | CHS_MAS | CHS_INS | MAS_INS |
| rs1801282 | 3 | 12368125 | *PPARG* | 2.20 x 10^-03^ | 6.50 x 10^-03^ | 6.04 x 10^-02^ | 3.90 x 10^-03^ | 4.80 x 10^-03^ | 3.20 x 10^-01^ |
| rs5215 | 11 | 17365206 | *KCNJ11* | 2.60 x 10^-03^ | 1.68 x 10^-02^ | 1.34 x 10^-02^ | 4.99 x 10^-01^ | 1.00 x 10^-04^ | 7.00 x 10^-04^ |
| rs7903146 | 10 | 114748339 | *TCF7L2* | 1.00 x 10^-04^ | 1.00 x 10^-04^ | 1.22 x 10^-01^ | 6.20 x 10^-01^ | 1.00 x 10^-04^ | 1.00 x 10^-04^ |
| rs10010131 | 4 | 6343816 | *WFS1* | 1.90 x 10^-03^ | 8.90 x 10^-03^ | 1.30 x 10^-03^ | 3.45 x 10^-01^ | 1.00 x 10^-04^ | 2.00 x 10^-04^ |
| rs757210 | 17 | 33170628 | *HNF1B (TCF2)* | 1.40 x 10^-03^ | 4.82 x 10^-01^ | 1.16 x 10^-02^ | 5.12 x 10^-02^ | 1.00 x 10^-04^ | 7.30 x 10^-03^ |
| rs10923931 | 1 | 120319482 | *NOTCH2* | 4.91 x 10^-02^ | 1.15 x 10^-01^ | 4.90 x 10^-03^ | 4.87 x 10^-01^ | 7.90 x 10^-03^ | 4.60 x 10^-03^ |
| rs7578597 | 2 | 43586327 | *THADA* | 1.00 x 10^-04^ | 1.00 x 10^-04^ | 8.52 x 10^-02^ | 3.89 x 10^-01^ | 1.00 x 10^-04^ | 1.00 x 10^-04^ |
| rs243021 | 2 | 60438323 | *BCL11A* | 1.00 x 10^-04^ | 1.00 x 10^-04^ | 1.20 x 10^-03^ | 4.58 x 10^-01^ | 1.00 x 10^-04^ | 6.00 x 10^-04^ |
| rs2943641 | 2 | 226801989 | *IRS1* | 1.00 x 10^-04^ | 2.00 x 10^-04^ | 4.74 x 10^-02^ | 1.27 x 10^-01^ | 1.00 x 10^-04^ | 3.00 x 10^-03^ |
| rs6780569 | 3 | 23173478 | *UBE2E2* | 2.80 x 10^-03^ | 9.50 x 10^-03^ | 8.90 x 10^-03^ | 4.63 x 10^-01^ | 2.59 x 10^-01^ | 1.31 x 10^-01^ |
| rs4607103 | 3 | 64686944 | *ADAMTS9* | 1.40 x 10^-03^ | 2.60 x 10^-03^ | 1.00 x 10^-04^ | 3.07 x 10^-02^ | 2.28 x 10^-02^ | 3.93 x 10^-02^ |
| rs1470579 | 3 | 187011774 | *IGF2BP2* | 5.80 x 10^-03^ | 3.42 x 10^-02^ | 5.07 x 10^-02^ | 4.53 x 10^-01^ | 2.90 x 10^-02^ | 1.14 x 10^-01^ |
| rs4457053 | 5 | 76460705 | *ZBED3* | 1.40 x 10^-03^ | 6.90 x 10^-03^ | 2.20 x 10^-03^ | 6.16 x 10^-01^ | 2.00 x 10^-04^ | 2.00 x 10^-04^ |
| rs7754840 | 6 | 20769229 | *CDKAL1* | 1.00 x 10^-04^ | 1.00 x 10^-04^ | 1.16 x 10^-02^ | 2.28 x 10^-01^ | 1.00 x 10^-04^ | 1.10 x 10^-03^ |
| rs864745 | 7 | 28147081 | *JAZF1* | 1.00 x 10^-04^ | 5.00 x 10^-04^ | 1.69 x 10^-01^ | 5.00 x 10^-04^ | 1.00 x 10^-04^ | 2.00 x 10^-04^ |
| rs972283 | 7 | 130117394 | *KLF14* | 1.46 x 10^-02^ | 1.04 x 10^-01^ | 2.61 x 10^-01^ | 1.40 x 10^-01^ | 1.10 x 10^-03^ | 1.14 x 10^-02^ |
| rs896854 | 8 | 96029687 | *TP53INP1* | 6.30 x 10^-03^ | 1.18 x 10^-02^ | 1.76 x 10^-01^ | 8.69 x 10^-02^ | 3.53 x 10^-01^ | 5.49 x 10^-02^ |
| rs13266634 | 8 | 118253964 | *SLC30A8* | 1.35 x 10^-02^ | 2.10 x 10^-02^ | 1.33 x 10^-02^ | 7.68 x 10^-02^ | 1.80 x 10^-01^ | 6.00 x 10^-04^ |
| rs10811661 | 9 | 22124094 | *CDKN2A/B* | 2.80 x 10^-03^ | 3.10 x 10^-03^ | 1.59 x 10^-01^ | 1.60 x 10^-03^ | 1.45 x 10^-02^ | 2.88 x 10^-02^ |
| rs13292136 | 9 | 81141948 | *CHCHD9* | 3.00 x 10^-03^ | 3.87 x 10^-01^ | 7.29 x 10^-02^ | 3.98 x 10^-02^ | 2.00 x 10^-02^ | 8.98 x 10^-01^ |
| rs17584499 | 9 | 8869118 | *PTPRD* | 1.64 x 10^-01^ | 8.84 x 10^-02^ | 3.20 x 10^-03^ | 6.12 x 10^-02^ | 2.00 x 10^-04^ | 1.00 x 10^-04^ |
| rs12779790 | 10 | 12368016 | *CDC123/CAMK1D* | 4.84 x 10^-02^ | 8.80 x 10^-02^ | 6.00 x 10^-04^ | 1.70 x 10^-03^ | 8.00 x 10^-04^ | 3.90 x 10^-02^ |
| rs1111875 | 10 | 94452862 | *HHEX/IDE* | 3.00 x 10^-04^ | 1.21 x 10^-02^ | 2.36 x 10^-01^ | 1.41 x 10^-01^ | 1.08 x 10^-01^ | 8.21 x 10^-01^ |
| rs2237892 | 11 | 2796327 | *KCNQ1* | 1.70 x 10^-03^ | 9.60 x 10^-03^ | 1.85 x 10^-02^ | 3.64 x 10^-01^ | 3.50 x 10^-03^ | 2.77 x 10^-01^ |
| rs231362 | 11 | 2648047 | *KCNQ1* | 2.59 x 10^-01^ | 7.15 x 10^-02^ | 3.30 x 10^-02^ | 1.00 x 10^-01^ | 1.00 x 10^-04^ | 1.82 x 10^-02^ |
| rs1552224 | 11 | 72110746 | *CENTD2* | 5.00 x 10^-04^ | 1.50 x 10^-03^ | 6.80 x 10^-02^ | 6.10 x 10^-03^ | 1.00 x 10^-04^ | 3.00 x 10^-04^ |
| rs10830963 | 11 | 92348358 | *MTNR1B* | 1.80 x 10^-03^ | 4.30 x 10^-03^ | 2.54 x 10^-02^ | 5.23 x 10^-02^ | 5.00 x 10^-04^ | 3.67 x 10^-02^ |
| rs1531343 | 12 | 64461161 | *HMGA2* | 3.03 x 10^-01^ | 1.04 x 10^-01^ | 7.47 x 10^-02^ | 2.01 x 10^-01^ | 3.00 x 10^-02^ | 1.30 x 10^-03^ |
| rs7961581 | 12 | 69949369 | *TSPAN8/LGR5* | 4.00 x 10^-04^ | 3.00 x 10^-04^ | 1.30 x 10^-03^ | 4.92 x 10^-02^ | 2.00 x 10^-04^ | 2.20 x 10^-02^ |
| rs7957197 | 12 | 119945069 | *HNF1A* | 4.00 x 10^-04^ | 1.10 x 10^-03^ | 1.17 x 10^-01^ | 3.31 x 10^-02^ | 1.00 x 10^-04^ | 1.00 x 10^-04^ |
| rs11634397 | 15 | 78219277 | *ZFAND6* | 6.50 x 10^-03^ | 2.73 x 10^-01^ | 1.60 x 10^-03^ | 5.54 x 10^-02^ | 2.00 x 10^-04^ | 1.57 x 10^-02^ |
| rs7172432 | 15 | 60183671 | *C2CD4A-C2CD4B* | 9.70 x 10^-03^ | 2.19 x 10^-02^ | 5.02 x 10^-02^ | 2.82 x 10^-01^ | 1.39 x 10^-02^ | 6.25 x 10^-02^ |
| rs8042680 | 15 | 89322341 | *PRC1* | 1.00 x 10^-03^ | 2.00 x 10^-04^ | 1.05 x 10^-01^ | 5.90 x 10^-03^ | 1.00 x 10^-04^ | 1.00 x 10^-04^ |
| rs9939609 | 16 | 52378028 | *FTO* | 2.00 x 10^-04^ | 1.40 x 10^-03^ | 1.84 x 10^-02^ | 2.47 x 10^-02^ | 1.00 x 10^-04^ | 5.55 x 10^-01^ |
| rs391300 | 17 | 2163008 | *SRR* | 6.80 x 10^-02^ | 6.26 x 10^-02^ | 2.22 x 10^-01^ | 6.98 x 10^-02^ | 3.07 x 10^-01^ | 5.12 x 10^-02^ |
| rs10425678 | 19 | 38669236 | *PEPD* | 5.00 x 10^-04^ | 1.74 x 10^-01^ | 2.20 x 10^-03^ | 1.27 x 10^-01^ | 2.00 x 10^-04^ | 3.00 x 10^-04^ |
