# Supplementary material for: Transferability of Type 2 Diabetes Implicated Loci in Multi-Ethnic Cohorts from Southeast Asia
Source: PLoS Genet. 2011 Apr 7;7(4):e1001363. doi: 10.1371/journal.pgen.1001363 (PMC3072366; doi:10.1371/journal.pgen.1001363)
Supplement: Table S5 — Number of samples excluded during quality control and their reasons for exclusion. Note that the same sample may be excluded for more than one reason and each sample falls into exactly one of the exclusion reasons. (0.05 MB DOC) [file pgen.1001363.s011.doc]

| Exclusion reason(s) | Number of samples removed | | | |
| --- | --- | --- | --- | --- |
|  | Chinese  Illumina 610 Quad | Chinese  Illumina 1Mduov3 | Malays | Indians |
| Missingness and/or Heterozygosity | 13 | 21 | 31 | 26 |
| Missingness and/or Heterozygosity and Gender Discrepancy | 4 | 11 | 6 | 8 |
| Cryptic Relatedness | 228 | 56 | 278 | 323 |
| Cryptic Relatedness and Gender Discrepancy | 1 | 2 | 1 | 3 |
| Population Structure | 24 | 17 | 163 | 39 |
| Population Structure and Gender Discrepancy | 2 | 3 | 7 | 0 |
| Gender Discrepancy | 24 | 31 | 44 | 16 |
| Cryptic Relatedness between the Chinese on the two genotyping arrays^*^ | 134 | 5 | NA | NA |
| High fasting glucose in the Controls | 144 | 18 | NA | NA |
| **Total** | 574 | 164 | 530 | 415 |

^*^ The denser Illumina1M array is preferentially retained over the Illlumia610 array.
