# Supplementary material for: Transferability of Type 2 Diabetes Implicated Loci in Multi-Ethnic Cohorts from Southeast Asia
Source: PLoS Genet. 2011 Apr 7;7(4):e1001363. doi: 10.1371/journal.pgen.1001363 (PMC3072366; doi:10.1371/journal.pgen.1001363)
Supplement: Text S1 — Description of results from the individual genome wide association studies (GWAS) and meta-analysis. (0.03 MB DOC) [file pgen.1001363.s012.doc]

In the Chinese GWAS, none of the SNPs achieved statistical evidence even at *P* < 10^-6^ (see **Table S1**), though two regions on chromosome 3 encompassing the *PEX5L* gene and a gene desert respectively exhibited evidence between 10^-6^ < *P* < 10^-5^. The Malay GWAS identified a region on chromosome 1 that is intronic to the pecanex-like 2 gene (*PCNXL2*), with the index SNP rs12027542 exhibiting an odds ratio (OR) of 1.41 (95% CI = 1.23 – 1.61, *P* = 4.33 × 10^-7^). Two other regions emerged with evidence in excess of *P* < 10^-5^, spanning the *CR2* gene on chromosome 1 and the *LPIN2* gene on chromosome 18 respectively (**Table S1**). Intriguingly, the latter gene has been implicated in mouse models for fatty liver lipodystrophy, which in human is associated with loss of body fat, insulin resistance and hypertriglyceridemia [1]. The Indian GWAS identified a SNP (rs1048886) intronic to a hypothetical protein (*C6orf57*) on chromosome 6 which exhibited genome-wide significance (OR = 1.54, 95% CI = 1.32 – 1.80, *P* = 3.48 × 10^-8^). The association at this SNP in the Malay GWAS trended in the same direction but was not statistically significant due in part to a lower risk allele frequency (OR = 1.21, 95% CI = 0.95 – 1.50, *P* = 8.23 x 10^-2^), but showed no evidence of T2D association in the Chinese (*P* = 0.995, see **Table S1**).

The meta-analysis of all three GWAS identified six regions with *P* < 10^-5^, including the well-established gene regions at *HHEX*/*KIF11* and *CDKAL1* that consistently replicated across multiple populations in the meta-analysis, and the region at *C6orf57* on chromosome 6 that was primarily driven by the evidence from the Indian GWAS. One of the remaining three regions stretches almost 400kb on chromosome 15 between 75.18Mb – 75.58Mb and spans the genes *HMG20A* and *TSPAN3*, with the risk allele at the top SNP (rs7119) displaying consistent evidence of T2D association in all three populations (single-population OR ranging between 1.22 and 1.28, meta-analysis OR = 1.24, 95% CI = 1.14 – 1.34, *P* = 5.24 × 10^-7^). Another region on chromosome 3 that is about 4kb downstream of *ZPLD1* similarly displayed consistent evidence across all three populations, with the single-population OR ranging between 1.15 and 1.24 at the top SNP (rs2063640) and the combined OR of 1.23 (95% CI = 1.13 – 1.34, *P* = 3.47 × 10^-6^). The last region on chromosome 21 displayed consistent evidence in both the Chinese and Indian GWAS, although a non-significant association trending in the same direction was observed in the Malays, yielding a combined evidence of *P* = 3.90 × 10^-6^ at the index SNP (rs2833610, meta-analysis OR = 1.17, 95% CI = 1.09 – 1.24) (see main text **Table 2** and **Table S2**).

References:

1. Peterfy M, Phan J, Xu P, Reue K (2001) Lipodystrophy in the fld mouse results from mutation of a new gene encoding a nuclear protein, lipin. Nat Genet 27: 121-124.
